# The Heart–Kidney Axis in Heart Failure and Chronic Kidney Disease: Mechanisms, Mediators, and Therapeutic Implications

**DOI:** 10.3390/biom16091241

**Published:** 2026-08-27

**Authors:** Aodi Fan, Xinwei Chen, Ke Yang, Xuefang Ma, Haohao Gao, Binyan Wang, Guanwei Fan, Lan Li

**Affiliations:** 1 State Key Laboratory of Modern Chinese Medicine, Key Laboratory of Pharmacology of Traditional Chinese Medical Formulae, Ministry of Education, Tianjin University of Traditional Chinese Medicine, Tianjin 301617, China; 2 National Clinical Research Center for Chinese Medicine Acupuncture and Moxibustion, State Key Laboratory of Component-Based Chinese Medicine, First Teaching Hospital of Tianjin University of Traditional Chinese Medicine, Tianjin 300193, China

**Keywords:** heart–kidney axis, CVD, HFpEF, CKD, cardiology

## Abstract

The heart–kidney axis has emerged as a central framework for understanding the bidirectional interactions that drive cardiorenal syndrome and broader cardiovascular–kidney–metabolic (CKM) disease. However, existing interpretations have often emphasized hemodynamic and neurohormonal mechanisms without fully integrating the growing evidence for endocrine, inflammatory, and metabolic mediators that coordinate injury across organs. This Review was therefore undertaken to provide an updated and clinically relevant synthesis of the physiological basis of heart–kidney communication, the mechanisms underlying its disruption, and the therapeutic implications of these insights. To achieve this aim, we performed a systematic narrative review of the literature using PubMed, Embase, Web of Science, and Scopus for studies, supplemented by manual screening of reference lists. Priority was given to original studies, large cohort analyses, randomized controlled trials, meta-analyses, and authoritative reviews. We integrated evidence spanning physiological regulation, maladaptive signaling pathways, emerging mediators, experimental models, and evolving treatment strategies. The reviewed evidence indicates that heart–kidney crosstalk is driven not only by altered perfusion and venous congestion, but also by sustained activation of the renin–angiotensin–aldosterone system (RAAS) and sympathetic nervous system (SNS), inflammation, oxidative stress, mitochondrial dysfunction, anemia, uremic toxins, and disordered mineral metabolism. Among novel mediators, fibroblast growth factor 23 (FGF23) emerges as a major bone-derived, chronic kidney disease (CKD)-associated endocrine mediator linking renal injury to cardiac hypertrophy, fibrosis, calcium mishandling, and diastolic dysfunction, whereas Klotho appears to exert counter-regulatory protective effects. Heart-derived natriuretic peptides, including atrial natriuretic peptide (ANP) and B-type natriuretic peptide (BNP), remain important modulators of renal blood flow, natriuresis, and volume homeostasis. We further highlight the translational relevance of newer biomarkers and therapies, including sodium–glucose cotransporter 2 inhibitors (SGLT2is), glucagon-like peptide-1 receptor agonists (GLP-1 receptor agonists), and mineralocorticoid receptor antagonists (MRAs), which may help address cardiac and renal dysfunction in parallel. Overall, this Review supports a revised conceptual model in which the heart–kidney axis is governed by multidirectional hemodynamic, neurohormonal, immune, and endocrine signaling networks. A more integrated understanding of these mechanisms may improve biomarker discovery, refine risk stratification, and promote therapies that target both organs simultaneously.

## 1. Introduction

Globally, the burden of cardiometabolic disease continues to rise and now represents a dominant threat to human health. Heart failure (HF), CKD, atherosclerotic cardiovascular disease (CVD), type 2 diabetes mellitus (DM) and obesity are increasingly recognized not as isolated disorders of single organs but as components of an interconnected systemic syndrome [1]. According to the American Heart Association, more than 64 million people worldwide are living with HF [2]. Among these patients, the prevalence of CKD exceeds 50%, whereas individuals with CKD have more than twice the risk of developing HF compared with those without renal disease [3]. Such a degree of co-occurrence is unlikely to be incidental and highlights the limitations of a single organ framework in cardiometabolic medicine.

The kidney has traditionally been viewed as an excretory organ responsible for waste elimination and for the regulation of fluid, electrolyte and acid–base balance. Over the past two decades, however, this concept has shifted substantially. The kidney is now understood to be a central regulator of systemic metabolism and endocrine signaling. In the fasting state, the kidney contributes approximately 20% of endogenous glucose production, of which glutamine-dependent gluconeogenesis accounts for about 10–20% of renal glucose release (equivalent to roughly 2–5% of total endogenous glucose production), thereby linking renal energy metabolism to acid–base homeostasis [4]. At the same time, approximately 90% of filtered glucose is reabsorbed in the early proximal tubule via sodium–glucose cotransporter 2 (SGLT2), with the remainder reabsorbed via sodium–glucose cotransporter 1 (SGLT1) in the distal proximal tubule, preserving systemic substrate availability. The kidney also directly influences cardiovascular biology through endocrine mediators such as erythropoietin, renin and Klotho. Erythropoietin (EPO) promotes mitochondrial biogenesis through endothelial nitric oxide synthase (eNOS) and protein kinase B signaling (PKB/Akt signaling), aligning myocardial mitochondrial capacity with systemic oxygen transport [5]. Klotho preserves myocardial redox homeostasis by limiting reactive oxygen species (ROS) generation and modulating NOD-like receptor pyrin domain-containing 3 (NLRP3) inflammasome-associated pyroptosis [6]. These advances have repositioned the kidney from a passive excretory organ to an active determinant of cardiovascular and metabolic homeostasis.

Against this background, the concept of the heart–kidney axis has emerged to describe the bidirectional and systemic network linking these two organs. This concept has three central features. The first is bidirectionality. CKD promotes cardiac remodeling and contributes to left ventricular hypertrophy, myocardial fibrosis and diastolic dysfunction, hallmarks of HF with preserved ejection fraction, through mechanisms that include uremic toxin accumulation, disruption of Fibroblast Growth Factor 23 (FGF23)-Klotho signaling and renal anemia [7]. Conversely, HF drives kidney injury through both reduced forward perfusion and venous congestion. Among these mechanisms, venous congestion appears especially important, as it promotes pericyte detachment, transition of pericytes to myofibroblasts and activation of platelet-derived growth factor receptor signaling, ultimately leading to renal interstitial fibrosis [8]. The second feature is systemic integration. The heart–kidney axis is not a simple exchange between two organs but a network involving the RAAS, the SNS, antidiuretic hormone (ADH), intermediary metabolism, inflammation and oxidative stress [9]. The third is metabolic relevance. From the accumulation of uremic metabolites in CKD to the reprogramming of myocardial energetics in HF, metabolic disturbance is a pervasive feature of heart–kidney crosstalk [10].

The stability of the heart–kidney axis is further shaped by three major upstream influences: circadian rhythms, inflammation and metabolic dysregulation. In both the kidney and the heart, cell autonomous molecular clocks regulate ion transport, RAAS activity and myocardial energy metabolism through transcriptional feedback circuits built around core clock genes. Disruption of these rhythms, whether due to aging, CKD, DM or shift work, impairs the normal circadian pattern of renal sodium and water excretion and blunts the expected nocturnal decline in blood pressure, thereby increasing the risk of cardiovascular events and progression to end-stage kidney disease [11]. Low-grade systemic inflammation constitutes a second major driver. In CKD, uremic toxins activate nuclear factor κB (NF-κB) signaling and promote the release of inflammatory mediators such as interleukin 6 (IL-6) and tumor necrosis factor, which directly injure cardiomyocytes and favor fibrosis [12]. In acute heart failure (AHF), pathological neurohormonal activation triggers parallel inflammatory pathways, establishing reciprocal inflammatory signaling between the heart and kidney. Metabolic dysregulation permeates the entire process, as impaired renal control of glucose, electrolytes and acid–base balance promotes myocardial energetic mismatch, electrophysiological instability and oxidative stress [1]. Importantly, these three factors are not independent. Circadian disruption can intensify inflammation, metabolic disturbance can impair rhythmic homeostasis, and inflammation can in turn further aggravate metabolic imbalance, creating a self-perpetuating cycle of cardiorenal dysfunction [13,14].

**Figure 1:**
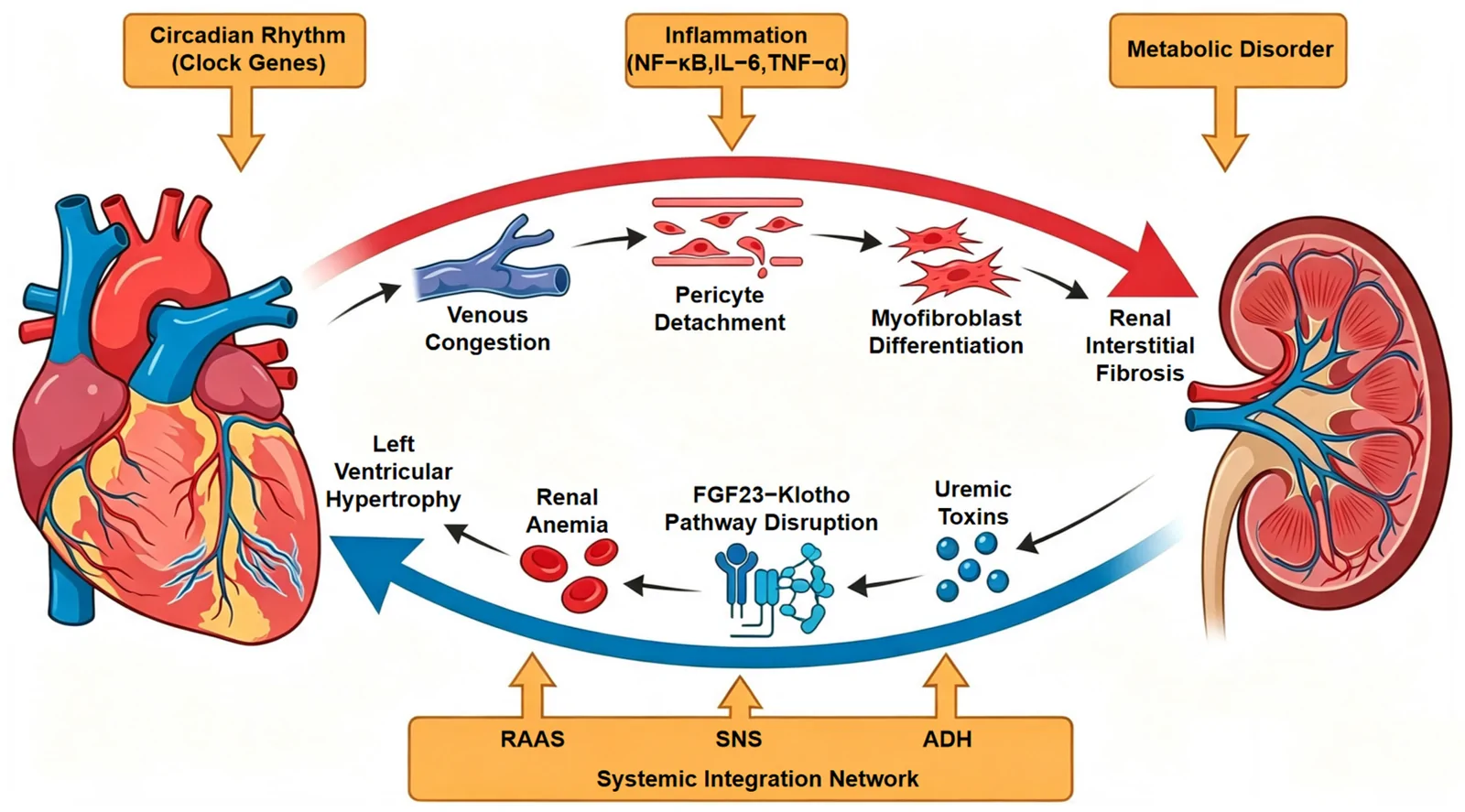
Bidirectional crosstalk between the heart and kidney. CKD drives cardiac injury via anemia, FGF23–Klotho dysregulation, uremic toxins, and neurohormonal activation. HF promotes renal injury through reduced perfusion, venous congestion, and pericyte-to-myofibroblast transition leading to interstitial fibrosis. Systemic integration, metabolic imbalance, inflammation, and circadian disruption further amplify this bidirectional pathological network.

In this Review, we summarize the physiological basis of the heart–kidney axis, the mechanisms that drive its disruption, emerging signaling mediators, experimental models and evolving therapeutic strategies. Our aim is to provide an integrated framework for both mechanistic investigation and clinical translation. A deeper understanding of the bidirectional dialogue between the heart and kidney may help redefine the conceptual and therapeutic landscape of cardiometabolic disease, support the identification of new biomarkers and targets, and guide interventions capable of addressing cardiac and renal dysfunction in parallel. Figure 1 illustrates the bidirectional heart–kidney interactions and their downstream effects.

## 2. Research Strategy

In this Review, we summarize the physiological basis of the heart–kidney axis, the mechanisms that drive its disruption, emerging signaling mediators, experimental models and evolving therapeutic strategies. Based on the objectives outlined above, we conducted a systematic narrative literature search in PubMed, Embase, Web of Science, and Scopus for articles published up to July 2026. Search terms included: “heart–kidney axis”, “cardiorenal syndrome”, “cardiovascular–kidney–metabolic syndrome”, “heart failure with preserved ejection fraction”, “chronic kidney disease”, “FGF23”, “Klotho”, “GDF15”, “natriuretic peptides”, “uremic toxins”, “SGLT2 inhibitors”, “GLP-1 receptor agonists”, “mineralocorticoid receptor antagonists”, and their combinations. We also manually screened the reference lists of key articles to identify any additional relevant studies. Priority was given to original research, large cohort studies, randomized controlled trials, meta-analyses, and authoritative reviews published in English. Our aim is to provide an integrated framework for both mechanistic investigation and clinical translation. A deeper understanding of the bidirectional dialogue between the heart and kidney may help redefine the conceptual and therapeutic landscape of cardiometabolic disease, support the identification of new biomarkers and targets, and guide interventions capable of addressing cardiac and renal dysfunction in parallel.

## 3. Physiological Interaction Between the Heart and Kidney

### 3.1. Hemodynamic Coupling: Bidirectional Interactions Between Cardiac Output and Renal Perfusion

The heart and kidneys are linked by a tightly integrated hemodynamic axis that is central to systemic homeostasis. By sustaining cardiac output and arterial pressure, the heart maintains renal plasma flow and glomerular filtration. Under physiological conditions, the kidneys receive ~20–25% of cardiac output, reflecting their high metabolic demand and essential role in fluid and solute handling. Despite their dependence on systemic perfusion, renal blood flow and glomerular filtration rate are buffered over a broad pressure range by autoregulatory adjustments in afferent and efferent arteriolar tone [15,16].

Conversely, the kidneys shape cardiovascular function through control of sodium and water balance, vascular volume and neurohormonal activity. By regulating natriuresis and diuresis, the kidneys determine venous return and cardiac preload. When renal perfusion falls, compensatory sodium and water retention may transiently support circulatory stability, but persistent volume expansion increases cardiac filling pressures and myocardial workload. Thus, heart–kidney hemodynamic coupling is inherently bidirectional: impaired cardiac function compromises renal perfusion, whereas renal dysfunction aggravates cardiac loading conditions and circulatory imbalance [17].

### 3.2. Neurohumoral Regulation: Bidirectional Modulation by the RAAS, SNS, and ADH

Under physiological conditions, the RAAS, the SNS and ADH form an integrated neurohumoral network that stabilizes arterial pressure, circulating volume and water–electrolyte balance [18]. Its function depends on tightly coordinated feedforward and feedback control, allowing rapid adaptation to changes in perfusion, volume status and sodium availability.

A key initiating event in this system is activation of renin-secreting juxtaglomerular (JG) cells. These specialized mural cells, located in the afferent arteriole, function as pressure-sensitive and neurohumoral transducers. Renin release is stimulated by three principal mechanisms: reduced stretch of the afferent arteriole (intrarenal baroreceptor mechanism), decreased sodium chloride delivery to the macula densa, and increased renal sympathetic nerve activity. Among these, sympathetic activation provides the most rapid link between systemic hemodynamic stress and humoral response. Norepinephrine released from postganglionic sympathetic terminals binds β1-adrenergic receptors on JG cells, activating Gs-protein-coupled adenylyl cyclase, increasing intracellular cyclic AMP (cAMP), and stimulating protein kinase A (PKA)-dependent exocytosis of renin granules as well as renin gene transcription. This cAMP-dominant signaling pathway is central to the secretory phenotype of JG cells. In parallel, reduced tubular NaCl delivery is sensed by macula densa cells through the apical Na^+^–K^+^–2Cl^−^ symporter (NKCC2), leading to increased production of prostaglandin E2 and nitric oxide, which act in a paracrine manner on adjacent JG cells to further enhance cAMP signaling and renin release [19]. Thus, neural, hemodynamic, and tubular signals converge at the level of the JG apparatus to determine the rate of renin secretion.

Released renin cleaves angiotensinogen to generate angiotensin I (Ang I), which is subsequently converted by angiotensin-converting enzyme (ACE) into angiotensin II (Ang II). As the principal effector of the cascade, Ang II exerts multiple downstream actions via AT1 receptor (AT1R)-dependent signaling pathways, including Gq protein (Gq)/phospholipase C (PLC)/inositol trisphosphate (IP3)/calcium ion (Ca^2+^) and protein kinase C (PKC), thereby inducing vasoconstriction, stimulating aldosterone secretion, constricting the efferent arteriole, and enhancing proximal tubular sodium reabsorption [20]. Importantly, Ang II also potentiates sympathetic outflow and norepinephrine release at both central sites, such as the circumventricular organs and nucleus tractus solitarius, and peripheral sites, including autonomic ganglia, the adrenal medulla, and presynaptic nerve terminals. In parallel, it stimulates the supraoptic and paraventricular nuclei of the hypothalamus to promote ADH release. ADH, in turn, increases water reabsorption in the collecting duct through V2 receptors and further supports vasoconstriction via V1a receptors, thereby amplifying the overall pressor response [21].

Despite this feedforward amplification, several intrinsic negative-feedback mechanisms constrain excessive activation. Ang II suppresses renin release through a short-loop feedback mechanism in JG cells by increasing intracellular Ca^2+^ via AT1R. ADH can also inhibit renin at the macula densa through V1a-dependent signaling. In addition, restoration of renal perfusion pressure and tubular NaCl delivery re-establishes tubuloglomerular feedback [22]. Beyond these mechanisms, the natriuretic peptide system, particularly ANP and BNP, is activated in response to increased cardiac filling and serves as a major counter-regulatory axis by dilating the afferent arteriole, suppressing renin and aldosterone secretion, and opposing the actions of ADH through cGMP-dependent signaling [23]. Moreover, the RAAS contains a protective angiotensin-converting enzyme 2 (ACE2)/Ang-(1–7)/Mas receptor axis that counterbalances AT1R-mediated vasoconstriction, oxidative stress, and profibrotic signaling [20].

Taken together, cardiovascular and renal homeostasis depends on the dynamic equilibrium among the SNS, RAAS, and ADH systems. Sympathetic input initiates renin release, Ang II amplifies both its own signaling and ADH activity, and multiple negative-feedback and counter-regulatory pathways prevent maladaptive overactivation, thereby ensuring stable perfusion of vital organs, particularly the brain.

### 3.3. Metabolic Crosstalk: Renal Regulation of Glucose, Electrolyte and Acid–Base Balance Shapes Cardiac Metabolism

The kidney influences cardiac metabolism not only through filtration but also by controlling systemic glucose availability, electrolyte gradients and acid–base balance, thereby providing a key metabolic interface within the cardiorenal axis. In the fasting state, the proximal tubule contributes substantially to endogenous glucose production through glutamine-dependent gluconeogenesis, while filtered glucose is reclaimed via SGLT2 and SGLT1 in a Na^+^/K^+^-ATPase-dependent manner [24]. These processes couple substrate handling to renal oxygen consumption and acid–base regulation. In CKD and diabetes, disturbed renal glucose handling and persistent nutrient excess promote maladaptive myocardial metabolic reprogramming, characterized by mTOR activation and suppression of AMP-Activated Protein Kinase (AMPK)/Sirtuin 1 (SIRT1)-Peroxisome Proliferator-Activated Receptor Gamma Coactivator 1-Alpha (PGC-1α) signaling, with consequent impairment of mitochondrial function, redox balance and metabolic flexibility [25].

Renal electrolyte handling also directly shapes cardiac electrophysiology. Distal tubular sodium and potassium transport, mediated by channels and transporters including the epithelial sodium channel (ENaC) and renal outer medullary potassium (ROMK) channel, determines the extracellular ionic milieu required for normal action potential generation and conduction. Impaired renal potassium excretion predisposes to hyperkalemia, which depolarizes the cardiomyocyte resting membrane potential, reduces sodium channel availability and slows conduction, thereby increasing the risk of malignant arrhythmias; conversely, hypokalemia prolongs repolarization and favors triggered activity [26].

Acid–base homeostasis provides a further mechanistic link. In proximal tubular cells, glutamine metabolism via glutaminase and glutamate dehydrogenase generates NH_4_^+^ for urinary acid excretion and new bicarbonate for systemic buffering. Although initially adaptive, sustained acid loading can activate intrarenal RAAS, endothelin-1 and complement signaling, promoting inflammation and fibrosis. Acid–base imbalance can affect vascular tone by altering vascular smooth muscle reactivity, sympathetic activity, and ventilatory status, thereby interfering with blood pressure regulation; for example, respiratory alkalosis can affect blood pressure through sympathetic excitation and induce arrhythmias. For the myocardium, metabolic or respiratory acidosis can depress myocardial contractility, whereas metabolic alkalosis is often accompanied by hypokalemia, which can disrupt myocardial electrical stability and increase the risk of arrhythmias. Acidosis generally reduces myocardial contractility because it affects multiple steps of excitation–contraction coupling, including inhibition of calcium influx and reduction of sarcoplasmic reticulum calcium release, thereby decreasing calcium delivery to the myofilaments. At the same time, acidosis reduces myofilament sensitivity to Ca^2+^, possibly by decreasing Ca^2+^ binding to troponin C and inhibiting maximal crossbridge force generation. Acidosis often causes K^+^ to shift from the intracellular to the extracellular compartment and also reduces K^+^ secretion and increases reabsorption in the collecting duct, thus predisposing to hyperkalemia; alkalosis enhances K^+^ secretion in the distal renal tubule, frequently leading to hypokalemia. Both hyperkalemia and hypokalemia can alter myocardial membrane potential and repolarization, increasing the risk of conduction abnormalities and arrhythmias [27,28,29]. In addition to these indirect effects, glutamine also exerts direct cardioprotective actions. Under oxidative stress, cardiomyocytes increase glutaminolysis, using glutamine-derived α-ketoglutarate for anaplerotic entry into the tricarboxylic acid cycle to support adenosine triphosphate (ATP) production and glutathione synthesis, thereby preserving cell viability. Glutamine therefore influences the heart not only indirectly through systemic substrate allocation and renal acid–base regulation but also directly through its roles in myocardial energy metabolism, antioxidant defense and mitochondrial function [30].

Taken together, by regulating the systemic supply and intertissue distribution of key metabolic substrates such as glucose and glutamine, preserving electrolyte balance, and maintaining acid–base homeostasis, the kidney continuously shapes cardiac metabolism and electrical stability. These tightly interconnected mechanisms sustain physiological heart–kidney integration, whereas disruption at any level may propagate through cardiorenal crosstalk and contribute to cardiovascular dysfunction.

### 3.4. Endocrine Function: Mechanistic Bidirectional Crosstalk Between Kidney-Derived EPO, Renin and Klotho and the Heart

In addition to its role in filtration, the kidney is an important endocrine organ in cardiovascular homeostasis. Through the production of EPO, renin and Klotho, the kidney participates in the regulation of myocardial energy metabolism, vascular tone, endothelial function and redox balance [31]. This relationship is not unidirectional, however, as changes in cardiac output, venous congestion, neurohormonal activation and systemic inflammation can in turn alter the renal synthesis of these factors. The endocrine heart–kidney axis should therefore be viewed as a dynamic bidirectional network rather than a simple kidney-to-heart pathway [32].

Among these mediators, EPO provides a direct link between renal oxygen sensing and cardiac metabolic adaptation. It is produced mainly by renal peritubular interstitial cells, where hypoxia stabilizes hypoxia-inducible factor 2-alpha (HIF-2α) and induces EPO transcription. Although EPO is traditionally considered in the context of erythropoiesis, its actions are not restricted to the bone marrow [33]. In the heart, activation of the EPO receptor triggers Janus Kinase 2 (JAK2)/Signal Transducer and Activator of Transcription 5 (STAT5) and PI3K/Akt–eNOS signaling, which has been associated with improved mitochondrial biogenesis, reduced oxidative stress, and inhibition of apoptosis, thereby supporting cardiomyocyte survival under stress conditions [34]. This protective axis may become blunted in HF. Reduced renal perfusion, venous congestion and inflammation can interfere with renal oxygen sensing and impair appropriate EPO production, contributing to cardiorenal anemia and further limiting myocardial oxygen supply [35].

Renin represents a second major endocrine pathway through which the kidney affects the cardiovascular system. Its release from juxtaglomerular cells is governed by three classic signals: reduced afferent arteriolar pressure, decreased NaCl delivery to the macula densa, and enhanced renal sympathetic stimulation via β1-adrenergic receptors. Once released, renin initiates the renin–angiotensin cascade, leading to generation of Ang II. Through angiotensin II type 1 receptor (AT1 receptor) signaling, angiotensin II activates downstream pathways including Gq/PLC/IP3/Ca2^+^, PKC, MAPK and NADPH oxidase, resulting in vasoconstriction, aldosterone secretion, sodium retention and amplification of sympathetic activity [36]. In parallel, local RAAS activity within the heart and vasculature contributes to the regulation of myocardial contractility, fibroblast activation and coronary vascular resistance [37]. Importantly, this pathway is highly sensitive to cardiac feedback. A fall in cardiac output reduces renal perfusion and increases baroreflex-mediated sympathetic drive, both of which stimulate renin release. What is initially compensatory may therefore become maladaptive, promoting persistent hemodynamic stress and adverse remodeling [38].

Klotho adds a further layer of complexity to this endocrine crosstalk. It is expressed predominantly in the distal nephron and exists in both membrane-bound and soluble forms, each with distinct but related functions. Membrane Klotho acts as a coreceptor for FGF23 by forming a receptor complex with FGFRs, thereby enabling FGF23 signaling in the kidney. This pathway promotes phosphate excretion and suppresses vitamin D activation and is therefore central to systemic phosphate homeostasis. By contrast, soluble Klotho exerts broader cardiovascular effects that are largely independent of FGF23 [39]. Experimental studies indicate that Klotho limits oxidative stress and inflammatory signaling, partly through suppression of insulin/insulin-like growth factor 1 (IGF-1), NF-κB and NLRP3 pathways, while preserving endothelial nitric oxide bioavailability and mitochondrial function [40]. Loss of renal Klotho disrupts these protective effects and also impairs the Klotho–FGF23 axis, favoring phosphate retention, endothelial dysfunction, vascular calcification and cardiac remodeling. Cardiac dysfunction may further suppress renal Klotho expression through congestion, neurohormonal activation and inflammatory stress, thereby creating a vicious cycle of progressive cardiorenal injury [41].

**Figure 2:**
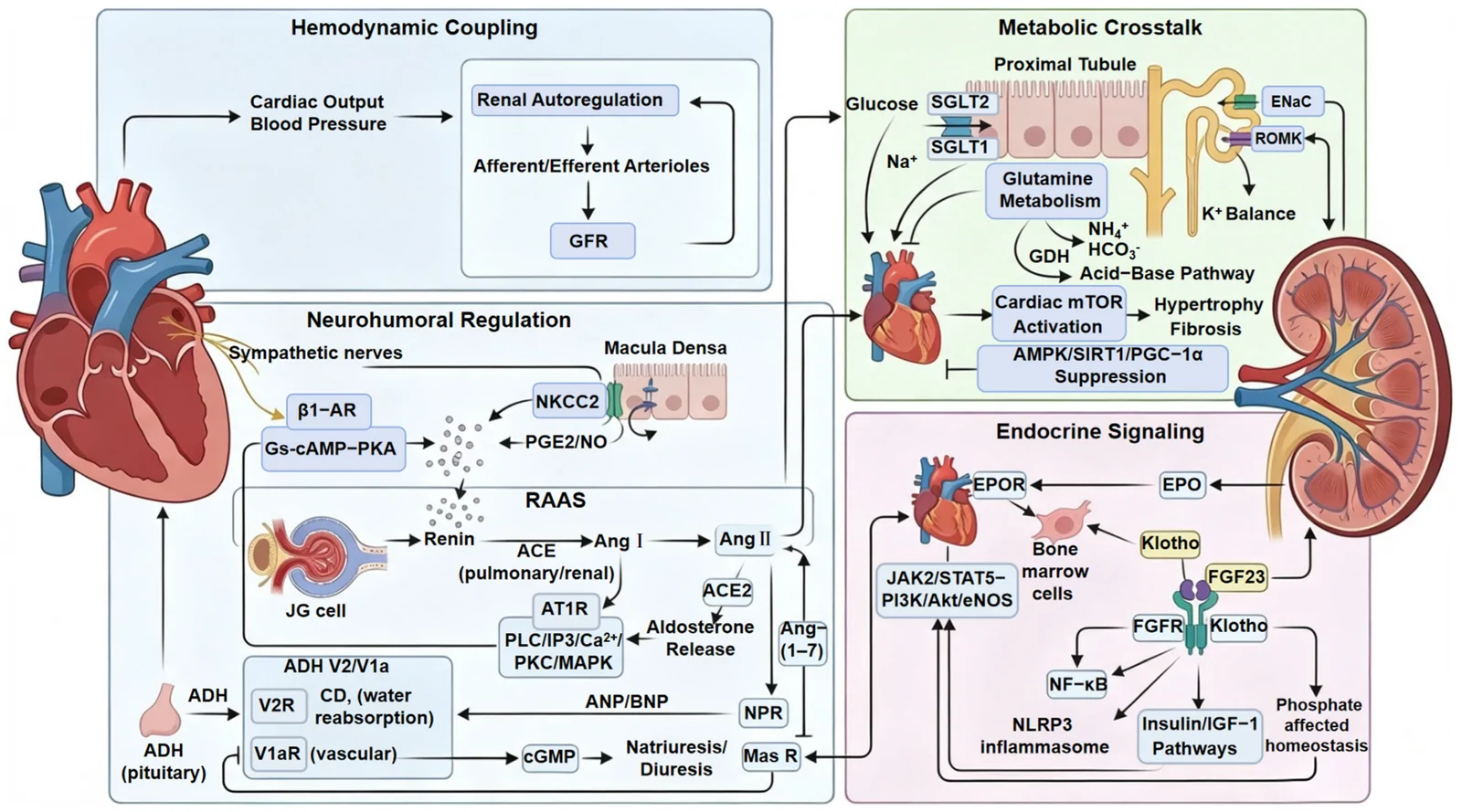
Schematic overview of physiological heart–kidney interactions. The figure illustrates the integrated hemodynamic, neurohumoral, metabolic, and endocrine mechanisms that sustain bidirectional crosstalk between the heart and kidney. Hemodynamic coupling links cardiac output and blood pressure to renal perfusion, which is buffered by afferent and efferent arteriolar autoregulation. Neurohumoral regulation is mediated through the RAAS, SNS, and ADH, with renin release triggered by β1−adrenergic stimulation, macula densa signals (NKCC2/PGE2/NO), and intrarenal baroreceptor sensing; Ang II/AT1R signaling downstream activates Gq/PLC/IP3/Ca^2+^/PKC/MAPK pathways, promoting vasoconstriction, aldosterone release, and sodium retention. Metabolic crosstalk involves proximal tubular glucose handling via SGLT2 and glutamine-dependent acid–base regulation (NH_4_^+^, HCO_3_^−^), while renal electrolyte and acid–base balance influence myocardial energetics and electrophysiology. Endocrine signaling is mediated by kidney-derived EPO (via EPOR–JAK2/STAT5−PI3K/Akt/eNOS), renin, and Klotho (via FGFR−FGF23 and insulin/IGF−1/NF−κB/NLRP3 pathways), which collectively regulate myocardial oxygen delivery, vascular tone, redox homeostasis, and mineral metabolism. These interconnected pathways form the physiological basis for heart−kidney integration, and their disruption contributes to cardiorenal dysfunction. Solid arrows indicate direct activation or promotion, whereas blunt-ended (flat-headed) arrows denote inhibition or blockade. (Abbreviations: ACE, angiotensin-converting enzyme; ADH, antidiuretic hormone; Ang II, angiotensin II; AT1R, angiotensin II type 1 receptor; eNOS, endothelial nitric oxide synthase; EPO, erythropoietin; EPOR, EPO receptor; FGF23, fibroblast growth factor 23; GDH, glutamate dehydrogenase; IGF-1, insulin-like growth factor 1; JG, juxtaglomerular; MAPK, mitogen-activated protein kinase; NF-κB, nuclear factor κB; NKCC2, Na^+^–K^+^–2Cl^−^ cotransporter 2; NO, nitric oxide; PGE2, prostaglandin E2; PI3K, phosphoinositide 3-kinase; PKA, protein kinase A; PLC, phospholipase C; PKC, protein kinase C; RAAS, renin–angiotensin–aldosterone system; SGLT2, sodium–glucose cotransporter 2; SNS, sympathetic nervous system).

Taken together, EPO, renin and Klotho illustrate how the kidney influences the heart through defined endocrine mechanisms involving oxygen sensing, hemodynamic control and mineral–metabolic signaling. At the same time, cardiac dysfunction feeds back on the kidney and reshapes the production of these mediators through hemodynamic, neural and inflammatory pathways. This reciprocal endocrine interaction is likely to be an important determinant of the transition from adaptive cardiorenal physiology to overt disease. These physiological interactions—hemodynamic, neurohumoral, metabolic, and endocrine—are summarized schematically in Figure 2, which illustrates how the heart and kidney operate as an integrated functional unit.

## 4. Impact of Kidney Disease on the Heart

### 4.1. CKD and Cardiovascular Risk

#### Epidemiological Evidence: CKD as an Independent Risk Factor for Cardiovascular Events

Among individuals with CKD, new-onset HF more often presents as heart failure with preserved ejection fraction (HFpEF) or mildly reduced ejection fraction than as HF with reduced ejection fraction, and the predominance of preserved ejection fraction becomes more marked as kidney function declines. Chronic kidney disease promotes HF through multiple, often overlapping pathways, including accelerated atherosclerosis, uremic cardiomyopathy, left ventricular hypertrophy, electrophysiological remodeling and arrhythmia. By contrast, once heart failure with reduced ejection fraction (HFrEF) develops, it is associated with the highest mortality risk [42].

This pattern has been consistently observed across cohorts. Among patients hospitalized with incident HF, HF with preserved or mildly reduced ejection fraction was more common than HF with reduced ejection fraction, and this difference was greater in those with CKD than in those without it [42]. The proportion of HF with preserved ejection fraction also increases with worsening kidney dysfunction, reaching particularly high levels in advanced CKD and dialysis populations [42]. Similar findings have been reported in the Chronic Renal Insufficiency Cohort study and in hemodialysis cohorts, in which preserved ejection fraction accounted for most new HF presentations [42,43]. Importantly, among patients with CKD, incident HFrEF carries a stronger association with mortality than incident HFpEF, whereas the association with progression to kidney failure requiring dialysis appears similar across HF phenotypes [42].

Several mechanisms probably explain the high burden of HF in CKD. Accelerated atherosclerosis is central, increasing the risk of ischemic heart disease, myocardial infarction and subsequent HF [44]. Outcomes after myocardial infarction are also substantially worse in CKD, with higher mortality that increases further as kidney function declines [45,46]. At the same time, CKD drives a distinct non-ischemic remodeling phenotype, often termed uremic cardiomyopathy, characterized by left ventricular hypertrophy, myocardial fibrosis and capillary rarefaction, all of which predispose to HF [44]. Additional contributors include atrial fibrillation and other electrophysiological disturbances, which interact bidirectionally with HF and further amplify risk [47]. The hemodynamic environment of CKD also favors adverse cardiac remodeling. Both preload and afterload are commonly increased. Volume expansion raises preload, whereas arterial stiffness, microvascular dysfunction, aortic stenosis and systemic hypertension increase afterload, each of which is more prevalent in CKD [44].

These abnormalities promote concentric remodeling, diastolic dysfunction and ultimately the HFpEF phenotype that is so common in this population.

Uremic cardiomyopathy develops progressively over the course of CKD and may begin before any major reduction in estimated glomerular filtration rate (eGFR) is clinically apparent [48]. In this setting, conventional left ventricular ejection fraction is often insensitive to early myocardial dysfunction. Speckle tracking echocardiography with strain analysis can detect subclinical abnormalities despite preserved ejection fraction and may therefore be particularly useful in type 4 cardiorenal syndrome [49]. In one study of 90 patients with CKD and 40 controls, longitudinal systolic strain as well as early and late diastolic strain rates were significantly impaired in CKD despite generally preserved ejection fraction [50]. In another cohort, global longitudinal strain independently predicted all-cause mortality [51]. Left ventricular hypertrophy is already present in about 30 to 40 percent of patients with kidney disease and rises to 70 to 90 percent at kidney failure, underscoring the cumulative cardiac burden of CKD [52].

Chronic kidney disease also worsens prognosis after myocardial infarction. Multiple studies have shown that reduced kidney function is associated with increased mortality after acute myocardial infarction across both older and broader postinfarction populations [53]. In a large contemporary study of 14,527 patients with acute myocardial infarction complicated by HF, left ventricular dysfunction or both, the risk of cardiovascular death increased progressively once eGFR fell below 81 mL per minute per 1.73 m^2^ [46]. As emphasized in accompanying commentary, eGFR provides a more reliable assessment of kidney function than serum creatinine alone because creatinine has a non-linear relation with glomerular filtration and is strongly influenced by age, sex and race [54].

Arrhythmia represents another major link between CKD and HF. The risk of both atrial and ventricular arrhythmias increases as kidney function declines. In the Atherosclerosis Risk in Communities study, among participants with glomerular filtration rate below 60 mL per minute per 1.73 m^2^, ectopic beats were present in more than 90 percent, ventricular tachycardia in 30.2 percent and atrial fibrillation in 7.4 percent [55]. RAAS activation after kidney injury drives sodium and water retention, volume overload and structural remodeling, all of which favor arrhythmia [56]. Uremic toxins further disrupt myocardial structure and conduction [57], and electrolyte disturbances, particularly hyperkalemia in advanced CKD and dialysis, can directly impair depolarization and cardiac conduction [58].

### 4.2. Renal Anemia and Cardiac Load

Renal anemia is a frequent complication of CKD. Although loss of functional nephron mass contributes, its central basis is impaired EPO production caused by defective renal oxygen sensing, which prevents an appropriate EPO response to tissue hypoxia [59,60]. In patients with coexisting HF and CKD, this defect is often compounded by functional EPO deficiency, in which responsiveness to EPO is reduced owing to receptor dysfunction and resistance within the bone marrow microenvironment [61]. The cardiovascular consequences are substantial. EPO deficiency lowers hemoglobin concentration and reduces blood oxygen carrying capacity, thereby limiting myocardial oxygen delivery. To maintain systemic oxygen supply, the circulation adapts by increasing cardiac output, creating a high output state that raises myocardial oxygen demand. This combination of reduced oxygen supply and increased oxygen demand provides the central hemodynamic link through which renal anemia promotes myocardial hypoxia and aggravates cardiac dysfunction [61,62]. Clinical studies have underscored the importance of this relationship. In end-stage kidney disease, each 1 g per dL decrease in hemoglobin has been associated with a 46 percent increase in the risk of left ventricular dilatation and a 28 percent increase in the risk of incident HF, supporting a direct role for anemia in increasing cardiac workload and driving structural remodeling [63].

Recent work has expanded this framework beyond hemodynamics alone. Disturbed iron handling in anemia can increase circulating FGF23, a bone-derived endocrine factor that has direct actions on the myocardium [64]. Mechanistically, FGF23 binds to fibroblast growth factor receptor 4 (FGFR4) on cardiomyocytes and activates prohypertrophic signaling, thereby contributing to increased left ventricular mass independently of blood pressure or volume status. Experimental studies further indicate that FGF23–FGFR4 signaling induces mitochondrial dysfunction and metabolic remodeling before overt structural abnormalities become apparent [65]. These effects likely impair myocardial energy homeostasis and increase susceptibility to contractile dysfunction. Thus, renal anemia promotes cardiac injury through two complementary mechanisms: a hemodynamic pathway driven by EPO deficiency, reduced oxygen carrying capacity and oxygen supply demand mismatch, and a non-hemodynamic pathway mediated by iron dysregulation, FGF23 excess, FGFR4-dependent hypertrophic signaling, mitochondrial dysfunction and metabolic remodeling. Together, these processes reinforce the vicious cycle of the cardiorenal anemia syndrome.

### 4.3. Uremic Toxins and Myocardial Toxicity

Indoxyl sulfate (IS) and p-cresyl sulfate (PCS) are among the best-characterized protein-bound uremic toxins. Because they are poorly removed by conventional dialysis, they accumulate in CKD and are increasingly recognized as direct mediators of cardiac injury, contributing to uremic cardiomyopathy and the broader cardiorenal syndrome [66,67]. A major effect of these toxins is the promotion of myocardial fibrosis through direct activation of cardiac fibroblasts and amplification of oxidative and inflammatory signaling. IS directly activates cardiac fibroblasts and induces time-dependent and dose-dependent increases in type I collagen, transforming growth factor β1 (TGF-β1) and α smooth muscle actin, consistent with a profibrotic phenotype [66]. At the signaling level, both IS and PCS activate apoptosis signal regulating kinase 1 (ASK1), which in turn stimulates the downstream p38 mitogen activated protein kinase (p38 MAPK) and extracellular signal regulated kinase 1/2 (ERK1/2) pathways. Activation of the ASK1–p38 MAPK/ERK1/2 axis promotes transcriptional programs linked to fibroblast activation, extracellular matrix production, myocardial hypertrophy and structural remodeling [67]. In parallel, IS upregulates cannabinoid receptor 1 (CB1) expression in cardiac fibroblasts and enhances Akt phosphorylation, thereby activating the CB1–Akt pathway and further promoting cardiac hypertrophy and fibrotic remodeling. Pharmacological blockade of CB1 attenuates these changes, supporting a causal role for this pathway in uremic cardiac injury [66].

In addition to their profibrotic effects, IS and PCS act as potent amplifiers of oxidative stress and inflammation. By activating nicotinamide adenine dinucleotide phosphate oxidase (NADPH oxidase), these toxins increase ROS generation and trigger NF-κB signaling [68,69]. This NADPH oxidase–ROS–NF-κB axis promotes direct cardiomyocyte injury, endothelial dysfunction and inflammatory activation, leading to increased expression of proinflammatory mediators such as IL-6 and tumor necrosis factor α (TNF-α). These mediators further amplify oxidative stress, inflammatory cell activation and myocardial damage, forming a self-reinforcing cycle of inflammation and remodeling. In patients with CKD, both free and total circulating levels of IS and PCS correlate independently with inflammatory indices, including IL-6 and TNF-α, as well as with markers of oxidative stress [68].

Experimental evidence further supports a causal role for these toxins in cardiac remodeling. In animal models, lowering circulating IS with the oral adsorbent AST-120 reduces myocardial transforming growth factor-β (TGF-β) expression, suppresses NF-κB activation and attenuates myocardial fibrosis [70].

**Figure 3:**
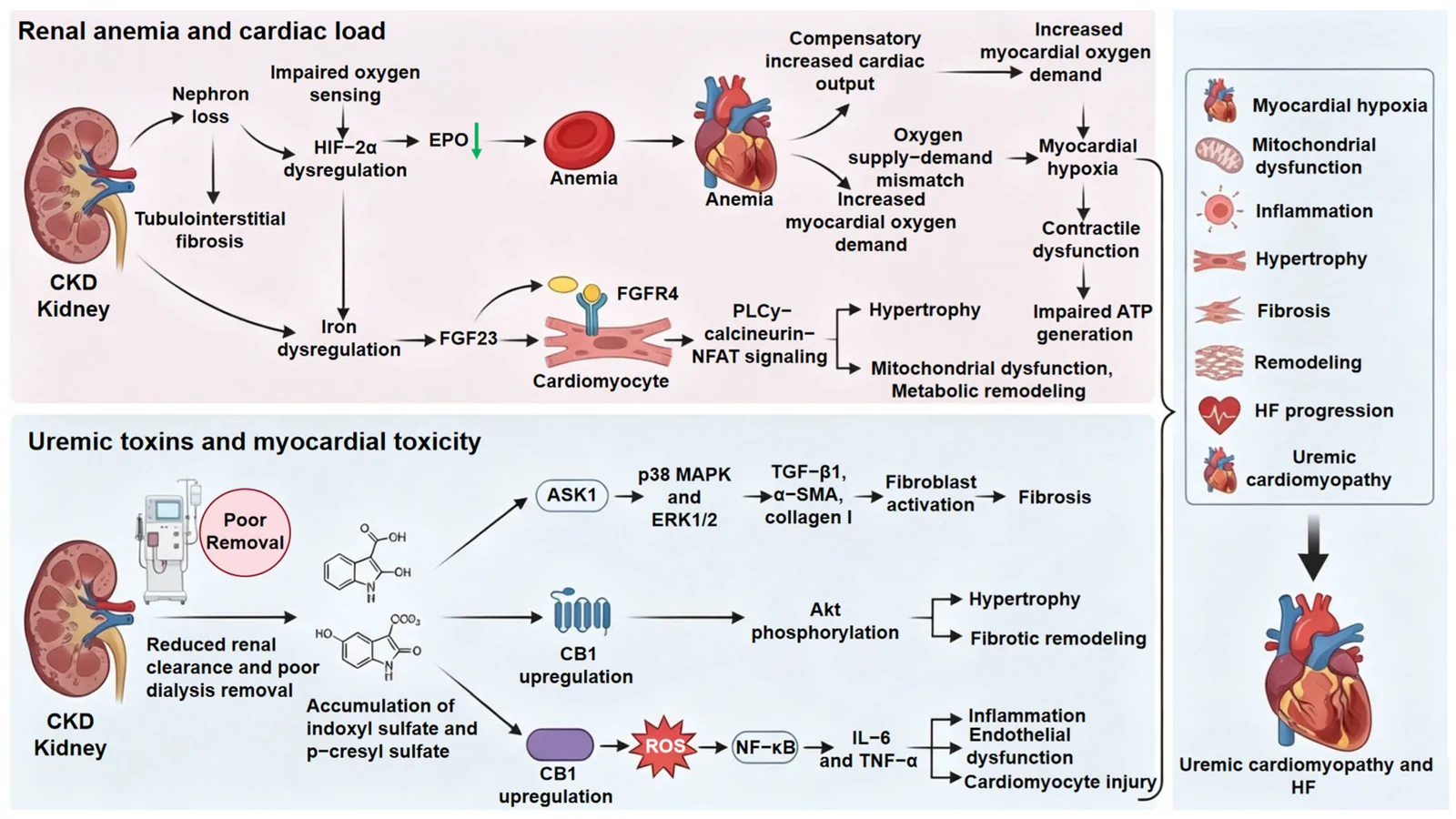
Schematic diagram of the mechanisms by which renal anemia and uremic toxins induce myocardial injury.

Taken together, these findings place IS and PCS at the center of a pathogenic network linking uremia to cardiac remodeling through three interrelated mechanisms: the ASK1–p38 MAPK/ERK1/2 profibrotic pathway, the CB1–Akt hypertrophic and fibrotic pathway, and the NADPH oxidase–ROS–NF-κB inflammatory oxidative stress pathway. Through these convergent signaling cascades, protein-bound uremic toxins drive fibroblast activation, extracellular matrix accumulation, cardiomyocyte injury, inflammation and progressive uremic cardiomyopathy. The integrated mechanisms by which renal anemia and uremic toxins drive cardiac damage in CKD are summarized in Figure 3. As shown, anemia and toxin accumulation act via complementary hemodynamic/non-hemodynamic routes and multiple profibrotic/proinflammatory signaling pathways, converging on the development of uremic cardiomyopathy and HF.

The mechanisms described above do not contribute equally across all stages of CKD, nor do they operate in isolation. Rather, they form a dynamic, stage-dependent hierarchy. FGF23–Klotho dysregulation appears relatively early, even in moderate CKD, and may act as an endocrine signal linking mineral disturbance to hypertrophy, though whether its cardiac effects are direct or merely reflect underlying bone–mineral disorder remains debated. Uremic toxin retention becomes increasingly dominant in advanced CKD, driving fibrosis and oxidative stress through ASK1–p38 MAPK/ERK1/2, CB1–Akt, and NADPH oxidase–NF-κB pathways; however, the independent contribution of individual toxins in humans is difficult to quantify, and their treatment responsiveness is uncertain. Renal anemia acts as an amplifier across moderate-to-advanced stages, contributing to myocardial hypoxia via both hemodynamic (EPO deficiency, oxygen mismatch) and non-hemodynamic (FGF23–FGFR4-mediated mitochondrial dysfunction) pathways, yet its causal role versus being a severity marker remains controversial, and clinical trials of anemia correction have shown inconsistent cardiovascular benefits. These pathways are not mutually exclusive: uremic toxins amplify inflammation and oxidative stress, which in turn enhance RAAS/SNS activation, while anemia exacerbates hemodynamic stress and further elevates FGF23, creating a self-reinforcing network. Recognizing this hierarchy may help refine risk stratification, guide biomarker selection, and inform the timing of combination therapies across the CKD trajectory. The relative importance and interplay of the above mechanisms across different CKD stages are summarized in Table 1.

Renal anemia reduces oxygen carrying capacity through EPO deficiency, leading to a compensatory increase in cardiac output and exacerbating oxygen supply–demand mismatch (hemodynamic pathway). Concurrently, disturbed iron metabolism upregulates FGF23–FGFR4 signaling, which induces mitochondrial dysfunction and metabolic remodeling (non-hemodynamic pathway). Uremic toxins (IS and PCS) act through three pathways—ASK1–p38 MAPK/ERK1/2, CB1–Akt, and NADPH oxidase–ROS–NF-κB—to directly activate cardiac fibroblasts, promote collagen deposition, and enhance oxidative stress and inflammatory responses. These two converging routes collectively lead to myocardial hypoxia, mitochondrial dysfunction, impaired contractile function, and impaired ATP production, eventually progressing to fibrosis, ventricular remodeling, uremic cardiomyopathy, and HF. Arrows denote promotion or progression relationships; green downward-pointing arrows indicate decreases/reductions. HIF-2α, hypoxia-inducible factor 2-alpha; CKD, chronic kidney disease; HF, heart failure; PLCγ, phospholipase C gamma; NFAT, nuclear factor of activated T cells; TGF-β1, transforming growth factor beta 1; α-SMA, alpha-smooth muscle actin; Akt, protein kinase B; NF-κB, nuclear factor kappa-B; ROS, reactive oxygen species; CB1, cannabinoid receptor 1; and ATP, adenosine triphosphate.

## 5. Impact of Cardiac Disease on the Kidney

### 5.1. HF and Cardiorenal Syndrome (CRS)

#### 5.1.1. Classification and Pathophysiological Mechanisms of CRS

The modern classification of cardiorenal syndrome was established by the Acute Dialysis Quality Initiative consensus conference in 2008 and remains widely used. According to the primary organ of onset and the temporal profile of injury, cardiorenal syndrome is divided into five subtypes: type 1, acute worsening of cardiac function leading to acute kidney injury (AKI); type 2, chronic cardiac dysfunction leading to progressive kidney injury; type 3, AKI provoking acute cardiac dysfunction or arrhythmia; type 4, CKD driving cardiac injury or dysfunction, including uremic cardiomyopathy; and type 5, systemic disorders such as sepsis, systemic lupus erythematosus or amyloidosis that simultaneously injure both organs [56,75] (Table 2).

The pathophysiology of cardiorenal syndrome is multifactorial and cannot be explained by hemodynamic disturbance alone. Classical models emphasized reduced cardiac output, diminished renal perfusion and compensatory activation of the RAAS, the SNS and arginine vasopressin, with consequent sodium and water retention, increased preload and progressive HF [73]. However, this framework is incomplete, as many patients develop cardiorenal dysfunction despite preserved ejection fraction or relatively maintained blood pressure [76]. A more integrated view places venous congestion alongside impaired forward flow as a major determinant of renal dysfunction. Increased central venous pressure causes renal venous hypertension, raises intrarenal vascular resistance and reduces the effective filtration gradient. Compensatory efferent arteriolar constriction may initially preserve filtration fraction, but this reserve is lost during more severe decompensation, with subsequent decline in glomerular filtration rate [77]. Right ventricular dysfunction can further impair renal perfusion by limiting left ventricular filling through ventricular interdependence and pericardial constraint [78]. At the same time, excessive neurohormonal activation not only increases vascular tone but also enhances proximal tubular sodium and water reabsorption, promoting oliguria and worsening congestion [79].

Non-hemodynamic mechanisms are equally important. Sustained activation of the SNS and the RAAS, together with inflammatory cytokines such as tumor necrosis factor, IL-1 and IL-6, directly impair myocardial and renal function; oxidative stress and disrupted nitric oxide signaling contribute to endothelial dysfunction [72], while FGF23 has been implicated in left ventricular hypertrophy in uremic cardiomyopathy [80]; congestive stretch can shift the vascular endothelium towards a proinflammatory phenotype [81]; and anemia may accelerate disease progression by reducing oxygen delivery and further stimulating neurohormonal activation [35]. Additional contributors include dendritic-cell-mediated immune responses [72], dysregulated adenosine and arginine vasopressin signaling, increased intra-abdominal pressure and the intrinsic vulnerability of the renal medulla to hypoxia [82].

Taken together, cardiorenal syndrome reflects the convergence of hemodynamic abnormalities, neurohormonal excess, inflammation, oxidative stress, endothelial injury and impaired oxygen delivery. This integrated model better captures the bidirectional and self-perpetuating nature of heart–kidney dysfunction.

**Table 2:** Clinical Subtypes of Cardiorenal Syndrome (CRS).

CRS Subtype	Course and Causal Direction	Description	Clinical Manifestations	Reference
Type 1	Acute, heart → kidney	HF leading to AKI	Cardiogenic shock and AKI	[83]
Type 2	Chronic, heart → kidney	Chronic heart failure leading to CKD	Chronic heart failure	[84]
Type 3	Acute, kidney → heart	AKI leading to AHF	Acute cardiac injury or arrhythmias	[85]
Type 4	Chronic, kidney → heart	Chronic kidney disease leading to chronic heart failure	Uremic cardiomyopathy	[75]
Type 5	Secondary cardiorenal syndrome	Systemic disorders simultaneously causing HF and kidney failure	Amyloidosis, sepsis, liver cirrhosis, systemic lupus erythematosus	[56]

#### 5.1.2. Prerenal Injury Due to Decreased Cardiac Output

Reduced cardiac output has long been regarded as the canonical mechanism of prerenal injury in type 1 cardiorenal syndrome. In this model, acute cardiac decompensation, such as acute decompensated HF, lowers effective arterial filling, reduces renal blood flow and ultimately decreases glomerular filtration rate [86]. When cardiac output falls to the range of cardiogenic shock, defined by a cardiac index below 1.8 L per minute per m^2^, or when mean arterial pressure drops below about 70 mmHg, renal autoregulation can no longer sustain filtration pressure [87]. The kidney responds by activating the RAAS through the juxtaglomerular apparatus. Ang II preferentially constricts the efferent arteriole, thereby helping to preserve intraglomerular hydrostatic pressure. At the same time, sympathetic overactivation constricts both afferent and efferent arterioles and increases renal vascular resistance [86]. Although these responses are intended to maintain perfusion of vital organs, they do so at the expense of the kidney and can promote renal ischemia.

This traditional framework, however, has been substantially revised by contemporary clinical data. In a post hoc analysis of the Evaluation Study of Congestive Heart Failure and Pulmonary Artery Catheterization Effectiveness (ESCAPE) trial, Nohria et al. reported that only right atrial pressure correlated weakly with baseline serum creatinine (r = 0.165, *p* = 0.03), while no baseline or change in hemodynamic parameters correlated with worsening renal function during hospitalization [88]. Subsequent studies have shown that renal venous hypertension can directly lower the renal perfusion pressure gradient and thereby reduce glomerular filtration, while renal interstitial edema raises intratubular pressure and further diminishes the transcapillary filtration gradient [89]. Thus, although low output remains relevant in states of profound hypoperfusion such as cardiogenic shock, venous congestion appears to be the dominant mechanism of renal dysfunction in most patients with acute decompensated HF [90]. This shift in perspective also helps explain why changes in renal function must be interpreted in a clinical context. During effective decongestive therapy, a fall in eGFR accompanied by a reduction in natriuretic peptide levels may be associated with improved outcomes. By contrast, an apparent improvement in eGFR in the absence of adequate decongestion may reflect more severe underlying disease and predict poorer survival [74]. These observations underscore the complexity of heart–kidney interactions and the limitations of a purely flow-based interpretation.

Even so, the pathophysiology of low-output prerenal injury remains important. Despite marked reductions in cardiac output, glomerular filtration may be transiently preserved through renal autoregulation and Ang-II-mediated efferent arteriolar constriction, which increase the filtration fraction. This adaptation, however, elevates peritubular capillary oncotic pressure and enhances proximal tubular sodium and water reabsorption. At the same time, reduced chloride delivery to the macula densa further stimulates the RAAS and SNS, promoting sodium and water retention along the nephron. Because the outer medulla depends on countercurrent exchange, it is especially susceptible to ischemia, and sustained renal hypoperfusion may accelerate nephron loss. In AHF, severe hypotension appears to correlate more closely with a decline in GFR than a numerical reduction in cardiac output alone. Once renal blood flow falls below a critical threshold, the compensatory increase in filtration fraction eventually fails [91].

#### 5.1.3. Venous Congestion and Renal Interstitial Fibrosis

Venous congestion is a key component of the backward failure pathway in cardiorenal syndrome and an important driver of renal interstitial fibrosis. Sustained elevation of central venous pressure impairs renal venous outflow, increases renal venous pressure, and reduces the renal perfusion pressure gradient, thereby promoting congestive kidney injury [92]. In experimental models, inferior vena cava ligation induces renal enlargement, medullary congestion, and progressive tubulointerstitial fibrosis [93]. Histologically, congestive nephropathy is characterized by dilatation of intrarenal veins and peritubular capillaries, relative preservation of glomerular structure, and mild tubular injury with interstitial fibrosis and tubular atrophy [94].

Mechanistically, renal venous hypertension increases postglomerular and interstitial hydrostatic pressure, causing intrarenal edema, tubular compression, and distortion of the medullary microcirculation [93,95]. This impairs oxygen diffusion in the hypoxia-prone outer medulla, leading to mitochondrial dysfunction and tubular stress [95]. Microvascular injury also promotes pericyte detachment from the vasa recta and peritubular capillaries, followed by transition to a myofibroblast phenotype and extracellular matrix deposition [93]. A central mediator of this response is platelet-derived growth factor receptor (PDGFR) signaling, which is markedly upregulated in congested kidneys; pharmacological inhibition of PDGFR signaling with imatinib substantially attenuates the increase in kidney weight and the extent of interstitial fibrosis in congested kidneys and suppresses TGF-β1-induced profibrotic signaling in human pericytes [93]. In parallel, inflammatory cell infiltration, increased intrarenal water content, and metabolic stress further amplify fibrogenesis [95].

Thus, venous congestion is not merely a passive hemodynamic abnormality but an active profibrotic stimulus that drives renal remodeling through medullary microvascular compression, hypoxic tubular injury, and PDGFR/TGF-β1-associated pericyte-to-myofibroblast transition.

#### 5.1.4. Impact of HF on the Kidney

In HFrEF, left ventricular and atrial dysfunction typically arise from common initiating disorders, including coronary heart disease, primary cardiomyopathy, hypertension and valvular heart disease. Impaired ventricular systolic and diastolic function, together with atrial dysfunction, reduces stroke volume and cardiac output, with consequent hypoperfusion of the kidneys and peripheral tissues [56]. These hemodynamic changes trigger compensatory activation of the SNS and the RAAS. Initially, these responses help preserve perfusion by increasing ventricular filling, supporting stroke volume and maintaining arterial pressure. Sympathetic activation also enhances myocardial contractility, raises systemic vascular resistance and increases left ventricular afterload. At the same time, reduced renal perfusion and heightened sympathetic tone stimulate renin release, leading to increased Ang II and aldosterone production. The result is sodium and water retention, expansion of intravascular volume and partial restoration of cardiac output. Vasopressin is likewise often increased, further promoting water retention and vasoconstriction [96].

In HF, however, these adaptive mechanisms become maladaptive. Renin release is no longer appropriately suppressed by volume expansion, and persistent renal sympathetic activation contributes to the inappropriate sodium and water retention that characterizes congestive HF [97]. Peripheral tissue hypoperfusion sustains sympathetic overactivity, which further drives renin secretion from the juxtaglomerular apparatus. Renin synthesis is also promoted by reduced hydrostatic pressure in the afferent arteriole and by diminished sodium delivery to the macula densa [98].

Together, these processes establish a self-reinforcing cycle of neurohormonal activation, volume overload and progressive cardiac and renal dysfunction.

#### 5.1.5. AHF and AKI

AKI in AHF results from the interplay of hemodynamic derangement and inflammatory injury. Type 1 cardiorenal syndrome should not be regarded solely as a consequence of reduced renal perfusion, but rather as a combined insult of forward hypoperfusion and backward venous congestion, with inflammatory signaling further amplifying tubular damage [99]. Although low cardiac output and renal hypoperfusion have traditionally been considered the major drivers of prerenal injury, reduced flow alone does not fully account for renal dysfunction. Glomerular filtration may be transiently preserved by renal autoregulation and Ang-II-mediated efferent arteriolar constriction through the AT1R–Gq/PLC/IP3/Ca^2+^ pathway. Overt renal dysfunction typically develops when mean arterial pressure falls below the autoregulatory threshold and renal perfusion pressure can no longer be maintained [91].

Venous congestion is now recognized as a major, and sometimes dominant, determinant of AKI in AHF. Elevated central venous pressure impairs renal venous outflow, increases renal interstitial hydrostatic pressure, and reduces the transglomerular filtration gradient, thereby promoting renal dysfunction independently of cardiac output. Renal venous hypertension also activates intrarenal RAAS, with Ang II/AT1R signaling stimulating NADPH oxidase (NOX)-derived ROS generation and upregulating endothelin-1 (ET-1), which together exacerbate microvascular dysfunction and tubular ischemia [56].

Beyond hemodynamic factors, neurohormonal and inflammatory activation constitutes a second major axis of injury. Excessive activation of the RAAS and the SNS initiates a proinflammatory cascade. Ang II–AT1R signaling activates NF-κB, leading to increased transcription of TNF-α, IL-1β, and IL-6. Elevated IL-6 further activates the interleukin 6 receptor (IL-6R)/glycoprotein 130 (gp130)–JAK/STAT3 pathway, which not only impairs myocardial contractility but also promotes renal injury by upregulating endothelial adhesion molecules intercellular cell adhesion molecule-1 (ICAM-1) and vascular cell adhesion molecule-1 (VCAM-1), facilitating leukocyte recruitment, and inducing tubular epithelial apoptosis [9]. In parallel, systemic hypoxia and oxidative stress activate hypoxia-inducible factor 1-α (HIF-1α), enhance proinflammatory gene expression, and drive macrophage polarization toward the M1 phenotype, thereby aggravating tubular injury through additional release of TNF-α and IL-1β [100].

Emerging evidence also highlights the contribution of innate immune signaling. Damage-associated molecular patterns (DAMPs), including high mobility group box 1 (HMGB1), activate Toll-like receptor 2 (TLR2)/Toll-like receptor 4 (TLR4) and downstream myeloid differentiation primary response 88 (MyD88)-dependent/-independent pathways, triggering tubular epithelial necroptosis and pyroptosis. Meanwhile, complement activation through complement components 3a/5a (C3a/C5a)–C5aR signaling promotes neutrophil and monocyte infiltration, further worsening tubulointerstitial injury [101]. Multiomics studies suggest that these inflammatory pathways are tightly linked to TGF-β1/Smad-mediated extracellular matrix remodeling and mitochondrial dysfunction, characterized by impaired respiratory chain activity and increased ROS production, forming an integrated heart–kidney injury network [102].

**Figure 4:**
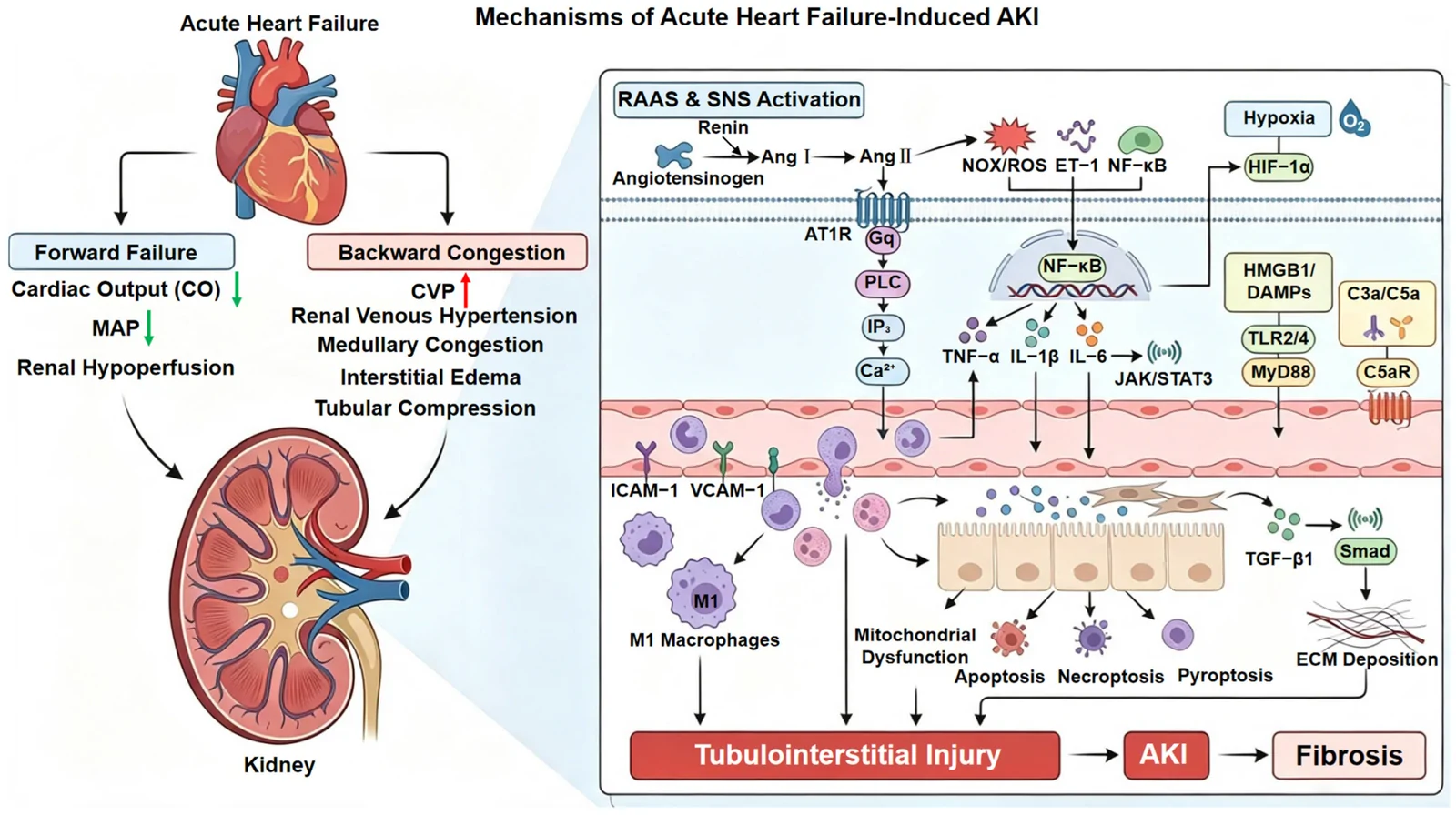
Schematic diagram of the mechanisms by which AHF induces AKI. AHF triggers AKI through three interconnected axes. Forward failure reduces cardiac output and mean arterial pressure, leading to renal hypoperfusion when autoregulatory capacity is exhausted. Backward congestion, driven by elevated central venous pressure, impairs renal venous outflow, increases interstitial hydrostatic pressure, and reduces the transglomerular filtration gradient. Concurrently, RAAS and SNS overactivation initiates a proinflammatory cascade via Ang II–AT1R signaling, which activates NF-κB and upregulates TNF-α, IL-1β, and IL-6; IL-6 further engages JAK/STAT3 to promote leukocyte recruitment and tubular apoptosis. Additional pathways include NOX/ROS-mediated oxidative stress, HIF-1α-driven inflammatory gene expression, TLR4/MyD88-dependent innate immune responses to DAMPs, complement C3a/C5a signaling, and TGF-β1/Smad-mediated extracellular matrix remodeling. These converging insults culminate in tubulointerstitial injury, characterized by M1 macrophage polarization, mitochondrial dysfunction, and multiple cell death modalities (apoptosis, necrosis, pyroptosis), ultimately leading to fibrosis and progressive renal dysfunction. Arrows denote causal or progression relationships. Red arrows indicate upregulation, activation, or increase; green arrows indicate downregulation, inhibition, or decrease. CO, cardiac output; MAP, mean arterial pressure; CVP, central venous pressure; RAAS, renin–angiotensin–aldosterone system; SNS, sympathetic nervous system; Ang II, angiotensin II; AT1R, angiotensin II type 1 receptor; Gq, Gq protein; PLC, phospholipase C; IP_3_, inositol trisphosphate; Ca^2+^, calcium ion; JAK/STAT3, Janus kinase/signal transducer and activator of transcription 3; NF-κB, nuclear factor kappa-B; NOX, NADPH oxidase; ROS, reactive oxygen species; ET-1, endothelin-1; HIF-1α, hypoxia-inducible factor 1-alpha; HMGB1, high mobility group box 1; DAMPs, damage-associated molecular patterns; TLR2/4, Toll-like receptors 2 and 4; MyD88, myeloid differentiation primary response 88; C5aR, complement C5a receptor; TNF-α, tumor necrosis factor-alpha; IL-1β, interleukin-1β; IL-6, interleukin-6; TGF-β1, transforming growth factor-β1; Smad, Smad protein; ECM, extracellular matrix; M1, M1 macrophages; and AKI, acute kidney injury.

Overall, AKI in AHF is driven by the convergence of forward failure, venous congestion, RAAS/SNS overactivation, and inflammatory signaling, with key mechanistic pathways involving AT1R–Gq/PLC/IP3/Ca^2+^, NOX/ROS, NF-κB, IL-6/JAK/STAT3, HIF-1α, TLR4/MyD88, and complement C3a/C5a signaling. These pathways interact through ROS, cytokines, and DAMPs to form a self-amplifying pathological network centered on tubular injury. The integrated mechanisms by which AHF drives AKI are summarized in Figure 4.

## 6. Role of Novel Mediators in the Heart–Kidney Axis

### 6.1. Endocrine and Circulating Mediators in Kidney–Heart Crosstalk

FGF23 is produced predominantly by osteocytes and osteoblasts rather than being a kidney-derived secretory factor. As an endocrine member of the fibroblast growth factor family, it is released into the circulation and acts on distant organs. Unlike paracrine fibroblast growth factors (FGFs), it is not sequestered within the extracellular matrix. Its canonical actions are mediated through fibroblast growth factor receptors together with the coreceptor Klotho in the kidney and parathyroid gland, where it reduces expression of sodium phosphate cotransporters in the proximal tubule, promotes urinary phosphate excretion and regulates vitamin D metabolism [103].

In CKD and cardiorenal syndrome, however, pathological elevation of FGF23 extends far beyond phosphate homeostasis and emerges as a central mediator of kidney-to-heart injury. Prospective studies in populations with and without CKD have consistently shown a dose-dependent association between higher FGF23 concentrations and increased risks of major cardiovascular events and death [71]. A proposed mechanism is that FGF23 may act directly on cardiomyocytes to promote pathological hypertrophy. Available experimental evidence suggests that this effect involves fibroblast growth factor receptor signaling and may occur independently of Klotho, although the molecular basis and clinical relevance of this non-canonical pathway remain to be fully established. [104]. In mice, administration of recombinant FGF23 induces concentric left ventricular hypertrophy within weeks, independently of blood pressure or renal function [104]. At the molecular level, FGF23 activates the calcineurin nuclear factor of activated T cells pathway, driving re-expression of fetal cardiac genes, including ANP, BNP and β-myosin heavy chain, and promoting cardiomyocyte growth and myocardial mass expansion [105].

FGF23 also amplifies cardiac injury indirectly through activation of the RAAS. In myocardial tissue, FGF23 increases expression of angiotensinogen and the Ang II type 1 receptor, thereby potentiating local Ang II signaling. This response promotes fibroblast activation and extracellular matrix deposition through the TGF-β Smad pathway, leading to interstitial fibrosis, increased ventricular stiffness and impaired myocardial compliance, which are key structural substrates of diastolic dysfunction [106]. Consistent with these effects, FGF23-driven hypertrophy and fibrosis contribute to progressive impairment of left ventricular relaxation and filling, a phenotype that maps closely onto HF with preserved ejection fraction [106].

Notably, even in early CKD, before severe loss of renal function is apparent, elevated FGF23 independently predicts left ventricular hypertrophy and diastolic dysfunction, suggesting that it may act upstream of more traditional drivers such as hypertension and volume overload [107]. In AKI, FGF23 also exerts direct cardiotoxic effects. Acute elevations in FGF23 enhance intracellular calcium transients and sarcoplasmic reticulum calcium release in cardiomyocytes through calcium–calmodulin-dependent protein kinase II signaling, resulting in systolic calcium overload, delayed diastolic calcium clearance and early electrical and mechanical instability. Experimental blockade of FGF23 signaling attenuates these calcium abnormalities and improves diastolic dysfunction in mouse models of AKI [108].

Clinical observations support this mechanistic framework. In patients across different stages of CKD, higher serum FGF23 levels correlate strongly with greater left ventricular posterior wall thickness and higher peripheral vascular resistance, whereas lower Klotho levels show the opposite pattern [109].

Collectively, these findings position FGF23 as a major effector of kidney to heart crosstalk, linking renal injury to cardiac hypertrophy, fibrosis, calcium dysregulation and ultimately diastolic dysfunction. It is important to interpret the above mechanistic findings with appropriate caution. While the epidemiological evidence linking elevated FGF23 to left ventricular hypertrophy, diastolic dysfunction and adverse cardiovascular outcomes is robust and consistent across multiple cohorts [71], several important caveats must be acknowledged. First, many experimental studies demonstrating direct Klotho-independent FGF23 effects on cardiomyocytes, including those using recombinant FGF23 administration, have employed concentrations that substantially exceed the physiological range in humans [104]. Whether such supraphysiological exposures translate into clinically relevant effects in patients remains uncertain. Second, elevated FGF23 in CKD rarely occurs in isolation; it is closely intertwined with disturbances in phosphate, calcium, parathyroid hormone, vitamin D, iron status and Klotho expression—all of which may independently contribute to cardiac remodeling [39]. Third, iron deficiency and inflammation are potent stimuli for FGF23 transcription and may confound the association between FGF23 and cardiac outcomes [61]. Finally, reduced renal Klotho expression in advanced CKD may not only impair FGF23’s canonical phosphate-regulating actions but also influence its non-canonical cardiac signaling through mechanisms that remain incompletely understood [39]. Thus, while FGF23 is a biologically plausible mediator of kidney-to-heart injury, the relative contributions of FGF23 per se versus its associated metabolic milieu to human cardiac pathology require further investigation. Prospective studies with serial measurements, Mendelian randomization analyses, and biomarker-stratified trials will be essential to distinguish causation from association and to determine whether therapeutic lowering of FGF23 confers cardiovascular benefit.

### 6.2. Heart-Derived Signaling Molecules

#### 6.2.1. BNP/ANP: Regulation of Renal Blood Flow, Diuresis, and Natriuresis

BNP and ANP are the prototypical heart-derived endocrine mediators of heart–kidney communication. By regulating renal hemodynamics, natriuresis, diuresis and volume homeostasis, these natriuretic peptides define a classic cardiac renal axis whose physiological and clinical relevance is supported by extensive epidemiological evidence [110]. This concept emerged from the seminal observation by de Bold and colleagues that the heart functions as an endocrine organ [111]. Subsequent work identified ANP, produced predominantly by the atria, and BNP, released mainly from the ventricles, particularly under pressure or volume overload, as the principal effectors of this axis [112]. Both peptides are synthesized in cardiomyocytes as inactive precursors and are processed into their active forms by the transmembrane serine proteases corin and furin before entering the circulation [113]. ANP and BNP act largely through their shared receptor, natriuretic peptide receptor A, which activates cyclic guanosine monophosphate signaling in target tissues, including the kidney [112]. In the kidney, these signals coordinate hemodynamic and tubular responses to promote sodium and water excretion. ANP and BNP increase glomerular filtration by dilating afferent arterioles and constricting efferent arterioles, thereby raising intraglomerular pressure [114]. They also inhibit tubular sodium reabsorption at multiple nephron segments, including the proximal tubule, the thick ascending limb and the collecting duct, through effects on Na^+^/K^+^-ATPase, the Na^+^/K^+^/2Cl^−^ cotransporter and epithelial sodium channels [115]. In parallel, they suppress the RAAS and SNS, reinforcing their natriuretic, vasodilatory and blood-pressure-lowering actions [116]. The clinical relevance of this natriuretic peptide pathway is especially evident in HF, a prototypical disorder of heart–kidney axis disruption. In the setting of HF, reduced renal perfusion and venous congestion trigger a cascade of neurohormonal activation, against which ANP and BNP act as essential endogenous counter-regulatory hormones to promote diuresis and natriuresis and maintain renal blood flow. The importance of this counter-regulatory system is underscored by the substantial disease burden of HF. HF affects an estimated 64.3 million people worldwide [117]. Given the sheer magnitude of this patient population, the impairment of the protective natriuretic peptide response often overwhelmed by sustained neurohormonal overdrive directly contributes to the refractory sodium and water retention that characterizes advanced HF. In this setting, BNP and ANP are not only established indicators of cardiac load but are also closely linked to renal function and prognosis. In a community-based cohort with 9 years of follow-up, N-terminal pro-B-type natriuretic peptide (NT-proBNP) independently predicted all-cause mortality and incident HF after adjustment for conventional risk factors, with stronger prognostic performance than ANP [118]. The importance of an intact natriuretic peptide pathway is further underscored by human genetic evidence. Individuals with homozygous loss of function variants in corin, who cannot efficiently generate active ANP, show reduced basal urinary electrolyte and creatinine excretion despite compensatory elevation of BNP, indicating that cardiac ANP is essential for normal renal homeostasis [119]. Additional support comes from studies in patients receiving dialysis, in whom plasma BNP and ANP are strong independent correlates of left ventricular mass and function, and higher concentrations are associated with markedly increased risks of all-cause and cardiovascular mortality [120]. Together, these mechanistic, genetic and epidemiological data establish BNP and ANP as both biomarkers and active effectors of heart–kidney crosstalk, with direct relevance to the development and progression of cardiorenal syndrome.

#### 6.2.2. Growth Differentiation Factor 15 (GDF15): Association with Kidney Function Decline and HF Prognosis

GDF15, a member of the TGF-β superfamily, is another heart-derived mediator with systemic effects. Although minimally expressed in the healthy adult heart, GDF15 is induced and released by cardiomyocytes under conditions of stress, including ischemia reperfusion, nitrosative stress, pressure overload, oxidative stress and inflammation [121]. Elevated circulating GDF15 levels are consistently associated with adverse outcomes in HF, indicating that this factor reflects both cardiac injury and broader systemic perturbation [122]. At present, however, experimental evidence supporting a direct pathogenic role of GDF15 in cardiorenal injury remains limited. Preliminary data from a type 2 diabetes mouse model suggest that GDF15 may be associated with enhanced NET formation and proinflammatory macrophage polarization and that pharmacological inhibition of GDF15 may be accompanied by attenuation of proteinuria, glomerulosclerosis, renal fibrosis, and cardiac dysfunction [123]. However, because these findings are derived primarily from a conference abstract, they should be regarded as preliminary and hypothesis-generating pending confirmation in full peer-reviewed studies. Importantly, the more established role of GDF15 at present is that of a stress-responsive circulating biomarker, whereas its canonical receptor biology is dominated by GDNF family receptor alpha-like (GFRAL)–rearranged during transfection (RET) signaling in the hindbrain; whether direct local actions of GDF15 occur in the heart, kidney, or immune compartments remains incompletely understood [123].

At the renal level, circulating GDF15 shows a robust inverse association with kidney function. In patients with chronic HF, GDF15 correlated negatively with eGFR and urinary sodium excretion, and in multivariable analysis it was the only factor independently associated with urinary sodium, whereas NT-proBNP was not, suggesting that GDF15 may more sensitively reflect impaired renal function and reduced natriuretic capacity [124]. Its prognostic value for kidney disease progression is also substantial. In a meta-analysis of 7813 patients with CKD, those in the highest GDF15 tertile had a 2.60-fold greater risk of kidney function decline than those in the lowest tertile, with a clear dose–response relationship [125].

GDF15 also provides strong prognostic information in HF. In AHF, increasing GDF15 levels were associated with a higher risk of all-cause death or HF rehospitalization within one year, and this association remained independent after adjustment for clinical variables and other biomarkers [126].

In older multimorbid patients with HFpEF, GDF15 was closely linked to renal insufficiency, anemia and inflammation and significantly improved the predictive performance of the Meta-Analysis Global Group in Chronic Heart Failure (MAGGIC) risk score [127]. Notably, its prognostic utility appears particularly relevant in the setting of concomitant renal dysfunction. In stable systolic HF with CKD, the predictive value of GDF15 exceeded that of BNP in multivariable models, and incorporation of GDF15 significantly improved risk reclassification, whereas this advantage was not evident in patients with preserved renal function [128]. Similarly, in CKD cohorts, higher GDF15 levels were independently associated with both all-cause mortality and incident HF, underscoring its role as an integrated biomarker of heart–kidney crosstalk [129].

#### 6.2.3. Mitsugumin 53 (MG53) and FSTL1: Emerging Cardio-Derived Factors Affecting Renal Metabolism

MG53, an E3 ubiquitin ligase released by cardiomyocytes and skeletal myocytes in response to hyperglycemia or hyperinsulinemia, is best known for its role in systemic insulin sensitivity [130]. Emerging evidence suggests that MG53 also confers renoprotection. In experimental settings, MG53 suppresses NF-κB signaling, thereby attenuating renal inflammation and fibrosis and slowing CKD progression. Conversely, MG53 deficiency exacerbates age-related and obstruction-induced renal injury and fibrosis [131].

FSTL1, also known as Transforming Growth Factor-β Stimulated Clone 36 (TSC-36) and Follistatin-Related Protein 1 (FRP), is a member of the follistatin family and is induced in the stressed heart, including in the settings of myocardial ischemia–reperfusion, pressure overload and myocardial infarction. Cardiomyocyte-derived FSTL1 limits pressure-overload-induced pathological hypertrophy, whereas systemic administration reduces ischemia–reperfusion injury by suppressing cardiomyocyte apoptosis and inflammation, consistent with autocrine and paracrine cardioprotective actions [132]. Notably, FSTL1 also appears to mediate heart-to-kidney protection. Heart-derived FSTL1 has been shown to attenuate glomerular hypertrophy, tubulointerstitial fibrosis and proteinuria, while reducing renal inflammation and oxidative stress. By contrast, cardiac FSTL1 deficiency aggravates kidney injury, whereas FSTL1 supplementation improves renal function, at least in part through AMPK activation and anti-inflammatory effects [132]. However, current evidence supporting FSTL1 in heart–kidney crosstalk is limited and derives mainly from preclinical studies, with scarce direct human data; thus, FSTL1 should be considered an emerging candidate mediator rather than an established mediator of the human cardiorenal axis.

### 6.3. Metabolites and Extracellular Vesicles (EVs)

#### 6.3.1. Heart–Kidney Axis Signaling Molecules Revealed by Metabolomics

Metabolomic profiling has identified uremic metabolites as an important class of signaling molecules within the heart–kidney axis. These metabolites accumulate with declining renal function, directly contribute to cardiac injury and, in parallel, drive metabolic remodeling in the kidney, thereby reinforcing a bidirectional pathological cycle [10]. Consistent with this concept, integrated proteomic and metabolomic analyses in a mouse model of pressure-overload-induced chronic HF showed that progressive cardiac dysfunction was accompanied by substantial renal metabolic remodeling after 12 weeks. In total, 66 differentially expressed proteins and 13 altered metabolites were identified, including significant changes in aspartic acid and isocitrate. These alterations were linked to perturbation of the phosphatidylinositol signaling system, glucagon signaling and glyoxylate and dicarboxylate metabolism, supporting the idea that cardiac injury can remotely induce mitochondrial dysfunction and disordered energy metabolism in the kidney [133].

#### 6.3.2. Role of EVs in Kidney–Heart Communication: miRNA and Protein Transport

Beyond soluble kidney-derived factors, small extracellular vesicles (sEVs) of renal cellular origin are emerging as potential mediators of cardiovascular injury in CKD. Under CKD conditions, circulating sEVs (~90 nm), isolated from platelet-poor and microvesicle-depleted plasma and positive for CD63, CD81, Alix, and flotillin-1, have been reported to carry cardiotoxic microRNAs such as miR-2110, miR-320b, and miR-484. These sEVs were linked to renal cellular sources, including tubular epithelial and endothelial cells, and after entering the circulation and being taken up by cardiomyocytes, their miRNA cargo can modulate genes involved in calcium handling, including phospholamban (PLN), and contractile function, including myosin heavy chain 6 (MYH6), thereby promoting cardiomyocyte apoptosis and contractile impairment and potentially contributing to CKD-associated HF. These vesicle-associated microRNAs may also have value as biomarkers of early, subclinical cardiac injury and could represent new therapeutic targets in chronic renocardiac disease. However, this field remains at an early stage, and further work is needed to define the causal contribution of kidney-derived sEVs to CVD and to determine whether they can be therapeutically targeted [134].

## 7. Role of Circadian Rhythm in the Heart–Kidney Axis

Circadian rhythm serves as a mechanistic integrator of the heart–kidney axis, operating through cell-autonomous molecular clocks that coordinate tissue-specific programs of transport, metabolism, neurohormonal responsiveness and electrophysiology over the 24 h cycle. At the core of this system lies a canonical transcriptional–translational feedback loop, in which the Circadian Locomotor Output Cycles Kaput (CLOCK): Brain and Muscle ARNT-Like 1 (BMAL1) heterodimer binds E-box elements to drive rhythmic transcription of Period (PER1–3) and Cryptochrome (CRY1–2) genes, while the accumulated PER/CRY complexes translocate to the nucleus and repress CLOCK:BMAL1 activity. This core oscillator is stabilized by an accessory nuclear receptor circuit, wherein REV-ERBα/β (NR1D1/2) represses and Retinoid-related Orphan Receptor alpha/beta/gamma (RORα/β/γ) activates BMAL1 transcription [135]. Although this molecular architecture is highly conserved across tissues, its downstream effectors differ substantially between renal tubular epithelial cells and cardiomyocytes, giving rise to organ-specific circadian outputs.

In the kidney, the intrinsic tubular clock governs nephron-segment-specific transport machinery, thereby shaping the circadian pattern of sodium and water handling. In the proximal tubule, clock-dependent transcriptional programs, particularly those involving PER1 and BMAL1, influence the expression and activity of sodium hydrogen exchanger 3 (NHE3), modulating sodium reabsorption and bicarbonate transport. In the thick ascending limb, rhythmic control of sodium–potassium chloride cotransporter 2 (NKCC2) contributes to time-of-day variation in medullary salt transport, whereas in the distal convoluted tubule, circadian regulation of sodium chloride cotransporter (NCC) affects sodium chloride reabsorption and blood pressure rhythmicity. In the collecting duct, clock components intersect with aldosterone-sensitive signaling to regulate ENaC subunits and coordinate water transport through Aquaporin 2 (AQP2), partly via temporal coupling with arginine vasopressin (AVP)–V2 receptor–cAMP/PKA signaling. Through these integrated mechanisms, the renal clock governs daily variation in natriuresis, diuresis, urinary concentrating capacity and extracellular volume balance, normally favoring greater sodium and water excretion during the active phase and relative retention during the rest phase [136]. In parallel, the renal clock interfaces with the intrarenal RAAS, contributing to oscillations in renin release, Ang II generation and aldosterone responsiveness, thereby linking clock function to pressure–natriuresis and systemic blood pressure homeostasis.

In the heart, the cardiomyocyte clock orchestrates temporal changes in substrate utilization, mitochondrial function, redox balance and electrophysiological stability. BMAL1 promotes metabolic flexibility by favoring glucose utilization and oxidation during the active phase, whereas REV-ERB family members contribute to rhythmic regulation of fatty acid metabolism and mitochondrial substrate preference during the rest phase [137]. The cardiac clock also modulates mitochondrial oxidative phosphorylation, antioxidant defense programs and ROS buffering capacity, reducing metabolic inefficiency during fluctuating workloads. In addition, circadian regulation extends to ion handling and electrical homeostasis, including genes involved in calcium cycling, membrane excitability and repolarization, which collectively shape diurnal variation in heart rate, contractility, conduction properties and arrhythmic susceptibility. At the integrated physiological level, these molecular rhythms contribute to normal day–night changes in cardiac output, vascular tone and autonomic balance, thereby supporting the physiological nocturnal decline in blood pressure of approximately 10–20% (the dipping pattern) [138].

Disruption of this circadian network—as occurs with aging, CKD, diabetes, shift work, sleep disturbance or intrinsic clock gene dysfunction—can destabilize both renal and cardiac homeostasis through convergent molecular and hemodynamic mechanisms. In the kidney, loss of rhythmic BMAL1/PER1-dependent transporter regulation blunts daytime natriuresis, alters nocturnal sodium and water handling, and impairs the normal timing of AVP-dependent AQP2 trafficking, thus contributing to nocturia [139]. Simultaneously, disruption of rhythmic intrarenal RAAS activity and impaired pressure–natriuresis favor a non-dipping or reverse-dipping blood pressure profile [138]. Sustained nocturnal hypertension increases cardiac afterload and, within the kidney, promotes glomerular hypertension, hyperfiltration, proteinuria and tubulointerstitial injury. At the signaling level, circadian misalignment may also amplify maladaptive SNS and RAAS activation, enhance Ang-II-dependent vasoconstrictive and sodium-retentive signaling, and increase oxidative stress and endothelial dysfunction. These perturbations may converge on downstream pathways relevant to progressive cardiorenal damage, including ROS accumulation, NF-κB-mediated inflammatory activation, altered endothelial nitric oxide bioavailability and TGF-β-driven profibrotic remodeling. In the heart, clock disruption impairs metabolic switching, compromises mitochondrial efficiency and destabilizes ion-channel expression, thereby increasing susceptibility to contractile dysfunction, electrical instability and adverse remodeling [137]. Once these abnormalities are established, a bidirectional vicious cycle may develop in which nocturnal hypertension, sodium retention, sleep disturbance, cardiac loading and renal injury reciprocally reinforce one another, ultimately accelerating cardiovascular events and progression toward end-stage kidney disease [140].

Despite these strong mechanistic links, the evidence supporting circadian dysregulation as a causal driver of cardiorenal disease differs markedly between experimental systems and human studies. In preclinical models, causal inference is relatively robust: genetic or tissue-specific perturbation of clock genes such as Bmal1, Clock, Per1 or Rev-erbα directly alters renal sodium transporter expression, blood pressure rhythmicity, myocardial metabolism and electrophysiological homeostasis, indicating that intrinsic clocks are functional determinants of organ physiology rather than passive markers of systemic timing. By contrast, in humans, most available evidence remains observational. Circadian disruption—manifested as shift work, chronotype misalignment, sleep fragmentation, non-dipping blood pressure or nocturia—is consistently associated with hypertension, cardiovascular events, CKD progression and adverse cardiorenal outcomes [138,140]. However, these associations must be interpreted cautiously. Residual confounding by obesity, diabetes, obstructive sleep apnea, medication timing, dietary sodium intake and socioeconomic factors remains difficult to exclude; reverse causation is also plausible, because established HF, CKD and nocturia can themselves disrupt sleep architecture, autonomic rhythmicity and hormonal circadian patterns. Moreover, many clinical studies rely on behavioral or hemodynamic surrogates of circadian disruption rather than direct measures of endogenous circadian phase or tissue clock function [141].

**Figure 5:**
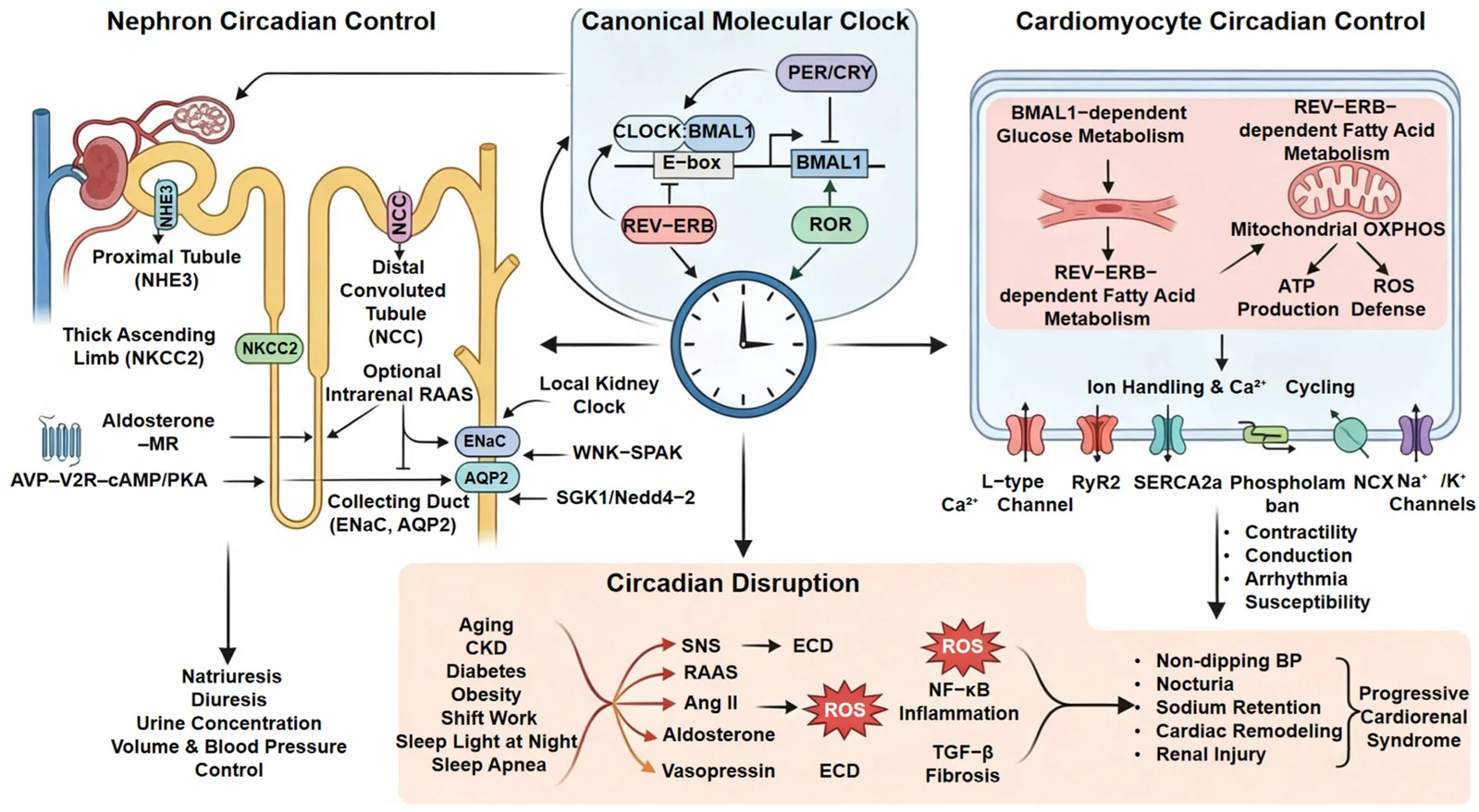
Schematic diagram of circadian control in the kidney and heart and the consequences of circadian disruption. The figure illustrates the molecular clock machinery and its tissue-specific outputs. In the kidney, the local tubular clock regulates segment-specific transporters (NHE3 in the proximal tubule, NKCC2 in the thick ascending limb, and ENaC/AQP2 in the collecting duct) through aldosterone–MR and AVP–V2R–cAMP/PKA signaling, thereby controlling natriuresis, urine concentration, and volume/BP homeostasis. In the heart, the cardiomyocyte clock governs BMAL1-dependent glucose metabolism, REV-ERB-dependent fatty acid oxidation, mitochondrial OXPHOS, ATP production, and ion handling (including Ca^2+^ cycling, SERCA2a, NCX, and Na^+^/K^+^ channels), which collectively modulate contractility, conduction, and arrhythmic susceptibility. Clock disruption—triggered by aging, CKD, diabetes, obesity, shift work, sleep disturbance, or light at night—activates SNS, RAAS, Ang II, and vasopressin, while promoting ROS generation and NF-κB/TGF-β-mediated fibrosis. These alterations lead to non-dipping BP, nocturia, sodium retention, cardiac remodeling, and progressive renal injury. Abbreviations: NHE3, sodium–hydrogen exchanger 3; NKCC2, sodium–potassium chloride cotransporter 2; MR, mineralocorticoid receptor; AVP, arginine vasopressin; V2R, vasopressin V2 receptor; cAMP, cyclic adenosine monophosphate; PKA, protein kinase A; ENaC, epithelial sodium channel; AQP2, Aquaporin 2; SGK1, serum- and glucocorticoid-inducible kinase 1; Nedd4-2, neural precursor cell expressed developmentally down-regulated protein 4-2; PER, Period protein; CRY, Cryptochrome; CLOCK, circadian locomotor output cycles kaput; BMAL1, brain and muscle ARNT-like 1; REV-ERB, reverse erythroblastosis virus α/β; ROR, retinoid-related orphan receptor; OXPHOS, oxidative phosphorylation; ATP, adenosine triphosphate; SERCA2a, sarco/endoplasmic reticulum Ca^2+^-ATPase 2a; NCX, sodium–calcium exchanger; NF-κB, nuclear factor kappa-B; ROS, reactive oxygen species; TGF-β, transforming growth factor-β; SNS, sympathetic nervous system; RAAS, renin–angiotensin–aldosterone system; Ang II, angiotensin II; BP, blood pressure; CKD, chronic kidney disease.

Thus, while biological plausibility is high and experimental evidence is compelling, direct proof that circadian dysregulation is an independent causal determinant of human heart–kidney disease remains incomplete. At present, circadian disruption is best regarded as a mechanistically credible and likely contributory factor in cardiorenal disease progression, whose causal importance in humans requires further validation through rigorous circadian phenotyping, longitudinal studies and interventional trials targeting temporal alignment. The molecular network of circadian regulation in the kidney and heart, and the consequent cardiorenal injury upon its disruption, is summarized in Figure 5.

## 8. Research Models

A broad and increasingly sophisticated repertoire of experimental platforms is now available to interrogate cardiorenal interactions. Rather than being viewed as competing approaches, these models should be considered complementary and synergistic, spanning reductionist systems to whole-organism and human-based investigations. Their thoughtful integration is critical for refining mechanistic insight and maximizing translational impact.

### 8.1. Animal Models

Despite their inherent inability to fully capture the biological heterogeneity of human disease, animal models remain indispensable for dissecting the systemic hemodynamic, inflammatory, fibrotic and metabolic mechanisms that underpin cardiorenal disorders.

In the context of CKD-associated cardiovascular injury, the 5/6 nephrectomy model remains one of the most widely used paradigms for mimicking nephron loss. This model recapitulates key features of progressive CKD, including proteinuria, hypertension, glomerulosclerosis, interstitial fibrosis, and both systolic and diastolic cardiac dysfunction. By contrast, the adenine-induced CKD model produces tubulointerstitial injury through intrarenal crystal deposition and is accompanied by hyperuricemia and cardiac remodeling; hypertension generally develops only after sustained dietary exposure [142].

For HFrEF, transverse aortic constriction (TAC) and permanent ligation of the left anterior descending (LAD) coronary artery remain the reference models for pressure-overload-induced hypertrophy and ischemic cardiomyopathy, respectively [143]. Pharmacologically induced models, including doxorubicin- and isoproterenol-based paradigms, provide useful complementary systems for studying cancer-therapy-related cardiotoxicity or adrenergic injury, although their translational fidelity is variable and context dependent [144].

In HFpEF and cardiorenal metabolic disease, there has been growing reliance on multihit models that combine genetic predisposition with environmental or metabolic stressors. Obese ZSF1 rats carrying mutant leptin receptor alleles spontaneously develop obesity, hypertension, insulin resistance and diastolic dysfunction and exhibit cardiac transcriptomic signatures that closely mirror those observed in human HFpEF [145]. Likewise, diet- and stress-based models such as high-fat diet combined with Nω-nitro-L-arginine methyl ester (L-NAME), a non-selective NOS inhibitor in rodents, or high-salt feeding combined with abdominal aortic constriction in pigs reproduce many cardinal features of the syndrome, including diastolic dysfunction, pulmonary congestion, renal fibrosis and systemic inflammation, thereby offering robust platforms for interrogation of the cardio–renal–metabolic axis [146].

### 8.2. Cellular Platforms, Organoids and Organ-on-a-Chip Systems

Cellular platforms are essential for high-resolution mechanistic interrogation. Human induced-pluripotent-stem-cell-derived cardiomyocytes (hiPSC-CMs), together with tubular and glomerular cell models, enable precise evaluation of cellular responses to uremic toxins, hemodynamic stress and inflammatory mediators. In parallel, heart–kidney coculture systems provide simplified but highly controllable settings in which to dissect paracrine signaling and metabolic crosstalk between the two organs. To more faithfully capture tissue architecture and multicellular complexity, three-dimensional systems have become increasingly important. hiPSC-derived kidney organoids provide tractable models of nephrogenesis and injury, whereas organ-on-a-chip platforms integrating fluid perfusion and mechanical stretch permit simulation of organ-level physiology and interorgan communication. Although these technologies continue to face limitations related to cellular maturation, standardization and long-term stability, they represent a substantial advance over conventional two-dimensional culture systems in terms of physiological relevance [147,148].

### 8.3. Human Tissue Studies, Longitudinal Cohorts and Multiomics Approaches

Ultimately, meaningful translation of mechanistic insights requires anchoring in human biology. Histopathological and molecular interrogation of explanted cardiac tissue, kidney biopsy specimens, and circulating or urinary biomarkers can directly illuminate disease endotypes and patterns of tissue remodeling. Deeply phenotyped longitudinal cohorts further enable reconstruction of disease trajectories over time and help define the temporal dynamics of cardiorenal decompensation. When coupled with multiomics technologies, including genomics, transcriptomics, proteomics and metabolomics, these human-centered platforms enable identification of novel molecular endotypes, predictive biomarkers and candidate therapeutic targets in cardiorenal syndrome. Although such approaches are predominantly associative, they generate clinically grounded hypotheses that can then be tested experimentally across complementary preclinical platforms [149].

## 9. Therapeutic Strategies: Targeting the Heart–Kidney Axis

### 9.1. Non-Pharmacological Interventions

Non-pharmacological interventions constitute the foundation of managing CKM syndrome. By optimizing behavioral and environmental factors, these interventions counteract the shared pathophysiological mechanisms driving cardiorenal disease, including oxidative stress, low-grade inflammation, insulin resistance, and lipid metabolic imbalance [150].

#### 9.1.1. Improvement of Cardiorenal Function by Time-Restricted Eating and Exercise

Intermittent fasting combined with exercise has been shown to attenuate diabetes-induced kidney injury and preserve renal histology, at least in part by modulating the polyol pathway, strengthening antioxidant defenses and suppressing inflammatory, apoptotic and TGF-β signaling [151]. In a 12-month study, time-restricted eating plus resistance training produced sustained improvements in inflammatory markers (IL-6, IL-1β and TNF-α), insulin sensitivity (fasting glucose, insulin and HOMA-IR) and lipid profile (total cholesterol, high-density lipoprotein and low-density lipoprotein), while also reducing overall cardiometabolic risk [152].

#### 9.1.2. Importance of Blood Pressure and Glycemic Control

Blood pressure and glycemic control remain foundational to cardiorenal risk reduction, and current guidelines increasingly frame both as central components of cardiorenal protective therapy. In the 2023 American Heart Association (AHA) staging framework for CKM syndrome, hypertension and diabetes are incorporated as core metabolic risk factors; stage 2 is defined by the presence of metabolic risk factors, including blood pressure ≥130/80 mmHg, hypertriglyceridemia, metabolic syndrome or diabetes, and/or CKD [150]. The 2025 AHA/American College of Cardiology (ACC) hypertension guideline retains 130/80 mmHg as the diagnostic and treatment threshold, with particular emphasis on earlier and more intensive control in patients with diabetes to reduce cardiovascular events, stroke, HF and progression of diabetic kidney disease [153]. Lifestyle intervention underpins glycemic management, including individualized nutrition therapy, regular physical activity, weight reduction and smoking cessation. These measures act synergistically with blood pressure and glucose control: weight loss improves insulin sensitivity and lowers blood pressure, exercise enhances endothelial function and metabolic homeostasis, and time-restricted eating may favor circadian alignment and autonomic balance. Together, these interventions target the core pathobiology of CKM syndrome and may slow the evolution of multiorgan damage [150].

### 9.2. Pharmacological Therapies

#### 9.2.1. SGLT2is

SGLT2is exert robust cardiorenal benefits through mechanisms that extend beyond glucose lowering. In the kidney, blockade of sodium–glucose cotransporter 2 in the proximal tubule increases urinary glucose and sodium excretion, restores tubuloglomerular feedback, reduces intraglomerular pressure and produces an initial eGFR dip that is associated with slower long-term decline and fewer renal events, including end-stage kidney disease [154]. At the cardiac level, SGLT2is improve myocardial energetics, with increased ketone body utilization, and reduce preload, filling pressures and wall stress; they may also confer cytoprotection through enhanced autophagic flux [155].

Clinical trials in diabetes, including Empagliflozin Cardiovascular Outcome Event Trial in Type 2 Diabetes Mellitus Patients (EMPA-REG OUTCOME), Canagliflozin Cardiovascular Assessment Study (CANVAS) and Dapagliflozin Effect on Cardiovascular Events–Thrombolysis in Myocardial Infarction 58 (DECLARE-TIMI 58), showed reductions in major adverse cardiovascular events and HF hospitalization [156]. In CKD, Canagliflozin and Renal Events in Diabetes with Established Nephropathy Clinical Evaluation (CREDENCE) and Dapagliflozin and Prevention of Adverse Outcomes in Chronic Kidney Disease (DAPA-CKD) confirmed substantial reductions in HF hospitalization and composite renal outcomes [157]. In HFrEF, Dapagliflozin and Prevention of Adverse Outcomes in Heart Failure (DAPA-HF) and Empagliflozin Outcome Trial in Patients with Chronic Heart Failure and a Reduced Ejection Fraction (EMPEROR-Reduced) demonstrated a 26% and 25% reduction in the composite of cardiovascular death or worsening HF, respectively (DAPA-HF: HR 0.74, 95% CI 0.65–0.85; EMPEROR-Reduced: HR 0.75, 95% CI 0.65–0.86), including in patients with concomitant CKD [158,159]. In HFpEF, Empagliflozin Outcome Trial in Patients with Chronic Heart Failure and a Preserved Ejection Fraction (EMPEROR-Preserved) and Dapagliflozin Evaluation to Improve the LIVEs of Patients with Preserved Ejection Fraction Heart Failure (DELIVER) similarly reduced HF hospitalization [160,161]. Together, these data make SGLT2is the only drug class with consistent benefit across the diabetes–CKD–HF continuum.

#### 9.2.2. GLP-1 Receptor Agonists

GLP-1 receptor agonists (GLP-1-RAs) provide cardiovascular benefit primarily through weight loss, glucose lowering, anti-inflammatory effects and attenuation of atherosclerotic burden. Their renal effects were initially thought to be largely reflected by reductions in albuminuria, but more recent evidence supports broader kidney protection in patients with diabetic CKD. Mechanistically, GLP-1-RAs promote natriuresis by inhibiting NHE3 in the proximal tubule and may also influence renal hemodynamics through nitric-oxide-dependent afferent arteriolar vasodilation [162]. In addition, these agents lower body weight and HbA1c, delay gastric emptying and reduce the risk of myocardial infarction and stroke through pleiotropic anti-inflammatory and antiatherosclerotic actions [163,164].

Clinical evidence from Liraglutide Effect and Action in Diabetes: Evaluation of Cardiovascular Outcome Results (LEADER), Semaglutide and Cardiovascular Outcomes in Patients with Type 2 Diabetes (SUSTAIN-6) and Researching Cardiovascular Events With a Weekly Incretin in Diabetes (REWIND) showed that GLP-1-RAs reduce major adverse cardiovascular events (MACEs) in patients with diabetes, with relative risk reductions of approximately 12–26%. By contrast, their effects on HF hospitalization have been modest and inconsistent, including in meta-analyses [165]. With regard to kidney outcomes, LEADER, REWIND and Effect of Efpeglenatide on Cardiovascular Outcomes (AMPLITUDE-O) demonstrated reductions in composite renal endpoints, driven largely by fewer cases of new-onset macroalbuminuria, whereas effects on eGFR slope were less consistent across studies [166]. Importantly, the FLOW trial subsequently showed that semaglutide reduced the risk of major kidney outcomes, substantial eGFR decline, kidney failure and cardiovascular death in patients with type 2 diabetes and CKD, providing robust evidence that GLP-1-RAs can confer clinically meaningful renal protection beyond albuminuria reduction. In patients with established HFrEF, however, the small LIVE and FIGHT trials did not show clinical benefit with liraglutide and raised safety concerns, including increased heart rate and a potential excess of arrhythmic events [167].

Thus, GLP-1-RAs are supported not only for patients with diabetes and ASCVD or elevated cardiovascular risk, particularly to prevent MACEs, but also for those with type 2 diabetes and CKD, in whom they may reduce both kidney disease progression and cardiovascular events, whereas their role in HF remains uncertain.

#### 9.2.3. Mineralocorticoid Receptor Antagonists (MRAs) and RAAS Blockers

MRAs and RAAS blockers are established cardiorenal protective therapies that act by dampening neurohormonal overactivation, limiting fibrosis and promoting natriuresis and diuresis. The clinical benefit of steroidal MRAs in HFrEF was established in Randomized Aldactone Evaluation Study (RALES), Eplerenone in Patients With Systolic Dysfunction After Myocardial Infarction (EPHESUS) and Eplerenone in Mild Patients Hospitalization And SurvIval Study in Heart Failure (EMPHASIS-HF), which showed reductions in all-cause mortality and HF rehospitalization [168]. In HFpEF, Treatment of Preserved Cardiac Function Heart Failure with an Aldosterone Antagonist (TOPCAT) did not meet its primary endpoint, but spironolactone reduced HF hospitalizations [169,170]. By contrast, the non-steroidal MRA finerenone exhibits a distinct receptor-binding profile, differential cofactor recruitment and favorable pharmacokinetics, including hepatic metabolism and a lower propensity for hyperkalemia in CKD [171]. In Finerenone in Reducing Kidney Failure and Disease Progression in Diabetic Kidney Disease (FIDELIO-DKD) and Finerenone in Reducing Cardiovascular Mortality and Morbidity in Diabetic Kidney Disease (FIGARO-DKD), finerenone reduced cardiovascular events and HF hospitalizations and slowed eGFR decline in patients with diabetes and CKD [172,173]. More recently, Finerenone Trial to Investigate Efficacy and Safety Superior to Placebo in Patients With Heart Failure (FINEARTS-HF) extended the evidence base for finerenone to patients with HF with mildly reduced or preserved ejection fraction, showing a reduction in worsening HF events and cardiovascular death, while the Finerenone in Heart Failure and Chronic Kidney Disease with Type 2 Diabetes (FINE-HEART) pooled analysis further supported benefit across a broad spectrum of HF phenotypes [174,175]. Collectively, these findings position finerenone as an important therapeutic option across the cardiorenal HF continuum. In addition, combination strategies may further enhance protection; notably, the ongoing COmbinatioN effect of FInerenone anD EmpaglifloziN in participants with chronic kidney disease and type 2 diabetes using a UACR Endpoint (CONFIDENCE) trial is directly evaluating finerenone plus empagliflozin in patients with CKD and type 2 diabetes. Further prospective studies are warranted to clarify the efficacy and safety of such combination approaches [176].

In addition to evidence from individual outcome trials, recent network meta-analyses have provided useful indirect comparative data for SGLT2is, GLP-1 receptor agonists, and finerenone in patients with T2DM and CKD, where head-to-head randomized trials remain lacking. In a network meta-analysis by Zhao et al., SGLT2is were associated with significantly greater reductions than finerenone in kidney function progression and hospitalization for HF, while the risks of MACEs, non-fatal myocardial infarction, stroke, cardiovascular death, and all-cause death were broadly similar between the two classes. Likewise, Zhang et al. compared finerenone, SGLT2is, and GLP-1 receptor agonists and found that SGLT2is conferred the greatest benefit for renal outcomes and HF hospitalization, whereas differences in MACEs, cardiovascular death, and all-cause mortality were less pronounced across the three drug classes. Collectively, these analyses support a pragmatic treatment framework in which SGLT2is are prioritized when CKD progression or HF risk predominates, GLP-1 receptor agonists are favored when atherosclerotic cardiovascular risk is the principal concern, and finerenone is considered an important complementary option for residual cardiorenal risk, particularly in patients with persistent albuminuric CKD despite standard therapy. Nevertheless, these findings are derived from indirect comparisons and should therefore be interpreted cautiously until confirmed by prospective head-to-head and combination-therapy trials [177,178].

## 10. Conclusions and Future Perspectives

The heart–kidney axis has important implications not only for understanding cardiorenal syndrome but also for the day-to-day management of CKM syndrome. Several practical messages emerge from the evidence reviewed here. First, cardiac and renal dysfunction should be assessed as a coupled process rather than as isolated organ disorders, because worsening congestion, decline in kidney function, anemia, and metabolic disturbance often reflect the same underlying pathobiology. Second, a normal or only mildly changing conventional renal or cardiac marker does not exclude active crossorgan injury; clinicians should interpret trajectories of renal function, albuminuria, natriuretic peptides, congestion status, and metabolic risk together. Third, treatment should be initiated early and guided by shared risk rather than by waiting for advanced failure of a single organ system. Fourth, residual risk in CKM syndrome is often driven by mechanisms beyond hemodynamics alone, including inflammation, fibrosis, mitochondrial dysfunction, and endocrine dysregulation, which may explain why some patients continue to progress despite standard therapy. Finally, effective management is likely to depend increasingly on integrated phenotyping, in which clinical variables are combined with mechanistically informative biomarkers to identify patients most likely to benefit from specific interventions.

In this context, the mediators discussed in this Review may be most valuable when used not simply as markers of disease severity but as tools for biological stratification. FGF23 is particularly relevant in patients with CKD-predominant disease, disordered mineral metabolism, left ventricular hypertrophy, and HFpEF-like remodeling and may help identify a subgroup in whom mineral–bone–cardiac signaling is a major driver of progression. GDF15 appears to reflect a broader stress response integrating inflammation, mitochondrial dysfunction, and tissue injury across both organs and may therefore be useful for recognizing patients with accelerated systemic vulnerability and poor resilience. EV-associated miRNAs are especially promising because they may capture active interorgan communication more directly than static circulating proteins; if standardized analytically, they could help define molecular endotypes that are not visible through routine clinical testing. A reasonable next step for the field is to embed these biomarkers prospectively into enrichment and stratification frameworks, so that future trials can test whether biomarker-defined subgroups show differential disease trajectories or treatment responses. Despite their promise, most circulating mediators discussed in this review are not yet clinically actionable biomarkers. For most candidates, the current evidence remains largely associative and insufficient to support routine implementation. Clinical translation will require a stepwise validation pathway, including assay standardization and analytical reproducibility, external validation across independent and diverse cohorts, demonstration of incremental value beyond established cardiorenal risk markers, assessment of temporal stability and clinically meaningful thresholds, and prospective testing within enrichment or stratified trial designs. Ultimately, a biomarker should be considered clinically ready only if it not only reflects pathobiology but also improves risk classification, informs treatment selection, and is linked to better patient outcomes.

These considerations are directly relevant to trial design. The next generation of CKM studies should move beyond single-drug, single-organ approaches and instead evaluate structured combination strategies with multiorgan endpoints. One pragmatic model would be to use SGLT2is as a therapeutic backbone, given their consistent benefits across HF and kidney disease, and then add GLP-1 receptor agonists in patients with obesity, diabetes, or prominent metabolic–inflammatory burden and non-steroidal mineralocorticoid receptor antagonists in those with albuminuric CKD, persistent fibrotic risk, or incomplete response to background therapy. Such sequencing is more clinically relevant than testing each class in isolation, because it better reflects how CKM syndrome is managed in practice and acknowledges that different pathways may dominate in different patients. Trial populations should therefore be defined not only by eGFR, albuminuria, or ejection fraction but also by trajectory-based and mechanism-informed characteristics, including rate of renal decline, recurrent congestion, biomarker profile, and metabolic phenotype.

Equally important is the choice of outcomes. Future studies would be strengthened by using hierarchical or composite endpoints that reflect the true multidimensional burden of CKM syndrome, such as cardiovascular death, HF hospitalization or urgent decompensation, sustained decline in eGFR, progression to kidney failure, change in albuminuria, and patient-reported functional status. Repeated measurement of FGF23, GDF15, natriuretic peptides, Klotho, and EV-miRNA signatures could be incorporated as mechanistic secondary endpoints to determine whether biological response parallels clinical benefit. In this setting, trajectory-based trial designs are especially attractive. Rather than classifying patients only at baseline, such designs would allow reassessment of risk over time and adaptation of therapy according to evolving cardiac and renal responses. This approach may be particularly valuable in CKM syndrome, where disease progression is rarely linear and where short-term stabilization of one organ does not always translate into long-term protection of the other.

In summary, the next step for the heart–kidney axis field is not simply to describe more mediators but to operationalize mechanistic knowledge into clinically actionable phenotyping and trial design. The most meaningful progress will come from linking biology to treatment selection, integrating combination therapies across organ systems, and adopting endpoints that reflect the true multidimensional burden of CKM syndrome. Such an approach offers the best opportunity to translate advances in heart–kidney biology into measurable benefit for patients.

## Figures and Tables

**Table 1:** Simplified stage-dependent hierarchy of kidney-to-heart injury mechanisms in CKD.

Mechanism	Main CKD Stage	Representative Mediators	Main Cardiac Phenotypes	Presumed Relative Contribution	Key Uncertainties	Reference
FGF23/Klotho dysregulation	Early to moderate CKD	FGF23, reduced Klotho, phosphate	LV hypertrophy, diastolic dysfunction, metabolic remodeling	Relatively more prominent in earlier CKD; persists later but rarely acts alone	Direct myocardial toxicity vs. marker of CKD-MBD; independence from phosphate burden remains debated	[39,71]
Uremic toxin retention	Moderate to advanced CKD	IS, PCS, TMAO	Fibrosis, oxidative stress, endothelial dysfunction, arrhythmogenic remodeling	Increases with CKD progression; likely dominant in advanced CKD	Difficult to quantify independent effect in humans; key pathogenic toxins and treatment responsiveness remain uncertain	[44,68]
Renal anemia	Moderate to advanced CKD	EPO deficiency/resistance, iron deficiency, hepcidin	Myocardial hypoxia, LV dilation/LV hypertrophy, HF progression	Moderate but clinically important; often acts as an amplifier	Causal role vs. disease severity marker; cardiovascular benefit of correction is inconsistent	[60,61,63]
Inflammation/oxidative stress	All stages, stronger in advanced CKD	IL-6, TNF-α, CRP, ROS, NADPH oxidase	Fibrosis, endothelial dysfunction, impaired relaxation	Persistent background mechanism that potentiates other pathways	Non-specific markers and limited proof of therapeutic targeting	[44,72]
RAAS/SNS activation	Early to advanced CKD	Ang II, aldosterone, norepinephrine	Hypertrophy, fibrosis, sodium retention, arrhythmia susceptibility	Foundational amplifier across stages	Difficult to separate maladaptive activation from compensation; residual risk despite blockade	[44,56]
Volume overload/hemodynamic stress	Moderate to advanced CKD	Sodium retention, plasma volume expansion, venous congestion	LV hypertrophy, atrial enlargement, diastolic dysfunction, HF decompensation	Major contributor in later CKD, especially for HF events	Chronic remodeling effects vs. acute congestion are not always clearly distinguishable	[73,74]

Abbreviations: CKD, chronic kidney disease; LV, left ventricular; HF, heart failure; RAAS, renin–angiotensin–aldosterone system; SNS, sympathetic nervous system; EPO, erythropoietin; MBD, mineral and bone disorder; ROS, reactive oxygen species; TMAO, trimethylamine N-oxide.

## Data Availability

The original contributions presented in this study are included in the article. Further inquiries can be directed to the corresponding author(s).

## Abbreviations

ACE, angiotensin-converting enzyme; ADH, antidiuretic hormone; AHF, acute heart failure; AKI, acute kidney injury; Akt, protein kinase B; AMPK, AMP-activated protein kinase; Ang I, angiotensin I; Ang II, angiotensin II; ANP, atrial natriuretic peptide; AQP2, Aquaporin 2; ASK1, apoptosis signal-regulating kinase 1; AT1R, angiotensin II type 1 receptor; ATP, adenosine triphosphate; AVP, arginine vasopressin; BMAL1, brain and muscle ARNT-like 1; BNP, B-type natriuretic peptide; cAMP, cyclic adenosine monophosphate; CB1, cannabinoid receptor 1; CKD, chronic kidney disease; CLOCK, circadian locomotor output cycles kaput; CRS, cardiorenal syndrome; CRY, cryptochrome; CVD, cardiovascular disease; DAMPs, damage-associated molecular patterns; DM, diabetes mellitus; eGFR, estimated glomerular filtration rate; ENaC, epithelial sodium channel; eNOS, endothelial nitric oxide synthase; EPO, erythropoietin; ERK1/2, extracellular signal-regulated kinase 1/2; ET-1, endothelin-1; EVs, extracellular vesicles; FGF23, fibroblast growth factor 23; FGFR, fibroblast growth factor receptor; FSTL1, follistatin-like 1; GDF15, growth differentiation factor 15; GFRAL, GDNF family receptor alpha-like; GLP-1, glucagon-like peptide-1; GLP-1-RAs, glucagon-like peptide-1 receptor agonists; HF, heart failure; HFpEF, heart failure with preserved ejection fraction; HFrEF, heart failure with reduced ejection fraction; HIF-1α, hypoxia-inducible factor 1-alpha; HIF-2α, hypoxia-inducible factor 2-alpha; HMGB1, high mobility group box 1; ICAM-1, intercellular cell adhesion molecule-1; IGF-1, insulin-like growth factor 1; IL-1β, interleukin-1β; IL-6, interleukin-6; IP_3_, inositol trisphosphate; IS, indoxyl sulfate; JAK2, Janus kinase 2; JG, juxtaglomerular; LAD, left anterior descending; L-NAME, Nω-nitro-L-arginine methyl ester; MACE, major adverse cardiovascular event; MAPK, mitogen-activated protein kinase; MG53, mitsugumin 53; MRAs, mineralocorticoid receptor antagonists; MyD88, myeloid differentiation primary response 88; NADPH oxidase, nicotinamide adenine dinucleotide phosphate oxidase; NCC, sodium chloride cotransporter; NF-κB, nuclear factor κB; NHE3, sodium–hydrogen exchanger 3; NKCC2, Na^+^–K^+^–2Cl^−^ cotransporter 2; NLRP3, NOD-like receptor pyrin domain-containing 3; NO, nitric oxide; NT-proBNP, N-terminal pro-B-type natriuretic peptide; p38 MAPK, p38 mitogen-activated protein kinase; PCS, p-cresyl sulfate; PDGFR, platelet-derived growth factor receptor; PER, period; PGC-1α, peroxisome proliferator-activated receptor gamma coactivator 1-alpha; PI3K, phosphoinositide 3-kinase; PKA, protein kinase A; PKC, protein kinase C; PLC, phospholipase C; RAAS, renin–angiotensin–aldosterone system; RET, rearranged during transfection; ROMK, renal outer medullary potassium channel; ROR, retinoid-related orphan receptor; ROS, reactive oxygen species; SERCA2a, sarco/endoplasmic reticulum Ca^2+^-ATPase 2a; sEVs, small extracellular vesicles; SGLT1, sodium–glucose cotransporter 1; SGLT2, sodium–glucose cotransporter 2; SGLT2i, sodium–glucose cotransporter 2 inhibitor; SIRT1, Sirtuin 1; Smad, smad protein; SNS, sympathetic nervous system; STAT3, signal transducer and activator of transcription 3; STAT5, signal transducer and activator of transcription 5; TAC, transverse aortic constriction; TGF-β, transforming growth factor-β; TLR2/4, Toll-like receptors 2 and 4; TNF-α, tumor necrosis factor-α; VCAM-1, vascular cell adhesion molecule-1.

## References

[1] Sebastian S.A., Padda I., Johal G. (2024). Cardiovascular-Kidney-Metabolic (CKM) syndrome: A state-of-the-art review. Curr. Probl. Cardiol..

[2] Palaniappan L.P., Allen N.B., Almarzooq Z.I., Anderson C.A.M., Arora P., Avery C.L., Baker-Smith C.M., Bansal N., Currie M.E., Earlie R.S. (2026). 2026 Heart Disease and Stroke Statistics: A Report of US and Global Data From the American Heart Association. Circulation.

[3] Núñez-Marín G., Santas E. (2025). Cardiorenal Disease and Heart Failure with Preserved Ejection Fraction: Two Sides of the Same Coin. Cardiorenal Med..

[4] Stumvoll M., Perriello G., Meyer C., Gerich J. (1999). Role of glutamine in human carbohydrate metabolism in kidney and other tissues. Kidney Int..

[5] Carraway M.S., Suliman H.B., Jones W.S., Chen C.W., Babiker A., Piantadosi C.A. (2010). Erythropoietin activates mitochondrial biogenesis and couples red cell mass to mitochondrial mass in the heart. Circ. Res..

[6] Wang S.S., Zhang X., Ke Z.Z., Zeng Y.X., Wen X.Y., Liu W.B., Zhao J., Zhuang X.D., Liao L.Z. (2025). Klotho attenuates D-galactose-induced cardiac aging through the ROS/NLRP3/pyroptosis pathway. J. Mol. Cell. Cardiol..

[7] Zannad F., Rossignol P. (2018). Cardiorenal Syndrome Revisited. Circulation.

[8] Matsuki T., Hirose T., Ohsaki Y., Shimada S., Endo A., Ito H., Takahashi C., Yamakoshi S., Oba-Yabana I., Anan G. (2022). Inhibition of platelet-derived growth factor pathway suppresses tubulointerstitial injury in renal congestion. J. Hypertens..

[9] Zhao B.R., Hu X.R., Wang W.D., Zhou Y. (2025). Cardiorenal syndrome: Clinical diagnosis, molecular mechanisms and therapeutic strategies. Acta Pharmacol. Sin..

[10] Curaj A., Vanholder R., Loscalzo J., Quach K., Wu Z., Jankowski V., Jankowski J. (2024). Cardiovascular Consequences of Uremic Metabolites: An Overview of the Involved Signaling Pathways. Circ. Res..

[11] Richards J., Gumz M.L. (2013). Mechanism of the circadian clock in physiology. Am. J. Physiol. Regul. Integr. Comp. Physiol..

[12] Huang S.Y., Chen Y.A., Chen S.A., Chen Y.J., Lin Y.K. (2016). Uremic Toxins—Novel Arrhythmogenic Factor in Chronic Kidney Disease—Related Atrial Fibrillation. Acta Cardiol. Sin..

[13] Festus I.D., Spilberg J., Young M.E., Cain S., Khoshnevis S., Smolensky M.H., Zaheer F., Descalzi G., Martino T.A. (2024). Pioneering new frontiers in circadian medicine chronotherapies for cardiovascular health. Trends Endocrinol. Metab..

[14] Panda S. (2016). Circadian physiology of metabolism. Science.

[15] Tonneijck L., Muskiet M.H., Smits M.M., van Bommel E.J., Heerspink H.J., van Raalte D.H., Joles J.A. (2017). Glomerular Hyperfiltration in Diabetes: Mechanisms, Clinical Significance, and Treatment. J. Am. Soc. Nephrol..

[16] Johannes T., Mik E.G., Nohé B., Unertl K.E., Ince C. (2007). Acute decrease in renal microvascular PO2 during acute normovolemic hemodilution. Am. J. Physiol. Ren. Physiol..

[17] Buliga-Finis O.N., Ouatu A., Badescu M.C., Dima N., Tanase D.M., Richter P., Rezus C. (2022). Beyond the Cardiorenal Syndrome: Pathophysiological Approaches and Biomarkers for Renal and Cardiac Crosstalk. Diagnostics.

[18] Abassi Z., Khoury E.E., Karram T., Aronson D. (2022). Edema formation in congestive heart failure and the underlying mechanisms. Front. Cardiovasc. Med..

[19] Yamaguchi H., Gomez R.A., Sequeira-Lopez M.L.S. (2023). Renin Cells, From Vascular Development to Blood Pressure Sensing. Hypertension.

[20] Lang C., Huang B., Chen Y., He Z. (2025). The role of the classical renin-angiotensin system and angiotensin-converting enzyme 2/Ang(1-7)/Mas axis in pulmonary fibrosis. Front. Med..

[21] Ameer O.Z. (2022). Hypertension in chronic kidney disease: What lies behind the scene. Front. Pharmacol..

[22] Kim S., Iwao H. (2000). Molecular and cellular mechanisms of angiotensin II-mediated cardiovascular and renal diseases. Pharmacol. Rev..

[23] Jangid M.K., Doshi G.M. (2025). Cross talk on therapeutic strategies: Natriuretic peptides and inhibiting neprilysin in hypertension management. Hypertens. Res..

[24] Lumpuy-Castillo J., Amador-Martínez I., Díaz-Rojas M., Lorenzo O., Pedraza-Chaverri J., Sánchez-Lozada L.G., Aparicio-Trejo O.E. (2024). Role of mitochondria in reno-cardiac diseases: A study of bioenergetics, biogenesis, and GSH signaling in disease transition. Redox Biol..

[25] Chai X.J., Gong W., Chen W., Min D.Y., Wang Y., Guan L. (2026). Metabolic reprogramming in chronic kidney disease-cardiovascular disease comorbidity: From molecular mechanisms to therapeutic strategies. Front. Pharmacol..

[26] Sinha F., Schweda F., Maier L.S., Wagner S. (2023). Impact of Impaired Kidney Function on Arrhythmia-Promoting Cardiac Ion Channel Regulation. Int. J. Mol. Sci..

[27] Orchard C.H., Kentish J.C. (1990). Effects of changes of pH on the contractile function of cardiac muscle. Am. J. Physiol..

[28] Urso C., Brucculeri S., Caimi G. (2015). Acid-base and electrolyte abnormalities in heart failure: Pathophysiology and implications. Heart Fail. Rev..

[29] Hamm L.L., Hering-Smith K.S., Nakhoul N.L. (2013). Acid-base and potassium homeostasis. Semin. Nephrol..

[30] Watanabe K., Nagao M., Toh R., Irino Y., Shinohara M., Iino T., Yoshikawa S., Tanaka H., Satomi-Kobayashi S., Ishida T. (2021). Critical role of glutamine metabolism in cardiomyocytes under oxidative stress. Biochem. Biophys. Res. Commun..

[31] Liu Y., Chen M. (2023). Emerging role of α-Klotho in energy metabolism and cardiometabolic diseases. Diabetes Metab. Syndr..

[32] McCallum W., Testani J.M. (2023). Updates in Cardiorenal Syndrome. Med. Clin. N. Am..

[33] Koury M.J., Haase V.H. (2015). Anaemia in kidney disease: Harnessing hypoxia responses for therapy. Nat. Rev. Nephrol..

[34] Burger D., Xenocostas A., Feng Q.P. (2009). Molecular basis of cardioprotection by erythropoietin. Curr. Mol. Pharmacol..

[35] Buliga-Finis O.N., Ouatu A., Tanase D.M., Gosav E.M., Seritean Isac P.N., Richter P., Rezus C. (2023). Managing Anemia: Point of Convergence for Heart Failure and Chronic Kidney Disease?. Life.

[36] Nádasy G.L., Balla A., Dörnyei G., Hunyady L., Szekeres M. (2024). Direct Vascular Effects of Angiotensin II (A Systematic Short Review). Int. J. Mol. Sci..

[37] Paul M., Poyan Mehr A., Kreutz R. (2006). Physiology of local renin-angiotensin systems. Physiol. Rev..

[38] Thakuri B., Das J.K., Roy A.K., Chakraborty A. (2023). Circulating renin-angiotensin systems mediated feedback controls over the mean-arterial pressure. J. Theor. Biol..

[39] Edmonston D., Grabner A., Wolf M. (2024). FGF23 and klotho at the intersection of kidney and cardiovascular disease. Nat. Rev. Cardiol..

[40] Prud’homme G.J., Wang Q. (2024). Anti-Inflammatory Role of the Klotho Protein and Relevance to Aging. Cells.

[41] Prud’homme G.J., Wang Q. (2026). Antiaging Properties of the Klotho Protein. Cells.

[42] Bansal N., Zelnick L.R., Scherzer R., Estrella M., Shlipak M.G. (2024). Risk Factors and Outcomes Associated With Heart Failure With Preserved and Reduced Ejection Fraction in People With Chronic Kidney Disease. Circ. Heart Fail..

[43] He J., Shlipak M., Anderson A., Roy J.A., Feldman H.I., Kallem R.R., Kanthety R., Kusek J.W., Ojo A., Rahman M. (2017). Risk Factors for Heart Failure in Patients With Chronic Kidney Disease: The CRIC (Chronic Renal Insufficiency Cohort) Study. J. Am. Heart Assoc..

[44] Noels H., van der Vorst E.P.C., Rubin S., Emmett A., Marx N., Tomaszewski M., Jankowski J. (2025). Renal-Cardiac Crosstalk in the Pathogenesis and Progression of Heart Failure. Circ. Res..

[45] Shroff G.R., Frederick P.D., Herzog C.A. (2012). Renal failure and acute myocardial infarction: Clinical characteristics in patients with advanced chronic kidney disease, on dialysis, and without chronic kidney disease. A collaborative project of the United States Renal Data System/National Institutes of Health and the National Registry of Myocardial Infarction. Am. Heart J..

[46] Anavekar N.S., McMurray J.J., Velazquez E.J., Solomon S.D., Kober L., Rouleau J.L., White H.D., Nordlander R., Maggioni A., Dickstein K. (2004). Relation between renal dysfunction and cardiovascular outcomes after myocardial infarction. N. Engl. J. Med..

[47] Sidhu B., Mavilakandy A., Hull K.L., Koev I., Vali Z., Burton J.O., Ng G.A. (2024). Atrial Fibrillation and Chronic Kidney Disease: Aetiology and Management. Rev. Cardiovasc. Med..

[48] Hayer M.K., Price A.M., Liu B., Baig S., Ferro C.J., Townend J.N., Steeds R.P., Edwards N.C. (2018). Diffuse Myocardial Interstitial Fibrosis and Dysfunction in Early Chronic Kidney Disease. Am. J. Cardiol..

[49] Gao X., Xiao W., Ji L., Li H., Zou A., Zhang X., Miao Z., Yang S., Yu S. (2024). Assessment of Left Ventricular Functional Impairment in Patients With Chronic Kidney Disease Using Three-Dimensional Speckle Tracking Imaging. Echocardiography.

[50] Hassanin N., Alkemary A. (2016). Early Detection of Subclinical Uremic Cardiomyopathy Using Two-Dimensional Speckle Tracking Echocardiography. Echocardiography.

[51] Krishnasamy R., Isbel N.M., Hawley C.M., Pascoe E.M., Leano R., Haluska B.A., Stanton T. (2014). The association between left ventricular global longitudinal strain, renal impairment and all-cause mortality. Nephrol. Dial. Transpl..

[52] Park M., Hsu C.Y., Li Y., Mishra R.K., Keane M., Rosas S.E., Dries D., Xie D., Chen J., He J. (2012). Associations between kidney function and subclinical cardiac abnormalities in CKD. J. Am. Soc. Nephrol..

[53] Kida H., Matsuoka Y., Sakamoto D., Hikoso S., Nakatani D., Okada K., Sunaga A., Sato T., Kitamura T., Sakata Y. (2025). Renal Function and its Time-Sensitive Influence on Survival Rates of Acute Myocardial Infarction. J. Am. Heart Assoc..

[54] Marx-Schütt K., Cherney D.Z.I., Jankowski J., Matsushita K., Nardone M., Marx N. (2025). Cardiovascular disease in chronic kidney disease. Eur. Heart J..

[55] Kim E.D., Soliman E.Z., Coresh J., Matsushita K., Chen L.Y. (2021). Two-Week Burden of Arrhythmias across CKD Severity in a Large Community-Based Cohort: The ARIC Study. J. Am. Soc. Nephrol..

[56] Rangaswami J., Bhalla V., Blair J.E.A., Chang T.I., Costa S., Lentine K.L., Lerma E.V., Mezue K., Molitch M., Mullens W. (2019). Cardiorenal Syndrome: Classification, Pathophysiology, Diagnosis, and Treatment Strategies: A Scientific Statement From the American Heart Association. Circulation.

[57] Yamagami F., Tajiri K., Yumino D., Ieda M. (2019). Uremic Toxins and Atrial Fibrillation: Mechanisms and Therapeutic Implications. Toxins.

[58] Montford J.R., Linas S. (2017). How Dangerous Is Hyperkalemia?. J. Am. Soc. Nephrol..

[59] Matsuoka T., Abe M., Kobayashi H. (2024). Iron Metabolism and Inflammatory Mediators in Patients with Renal Dysfunction. Int. J. Mol. Sci..

[60] Babitt J.L., Lin H.Y. (2012). Mechanisms of anemia in CKD. J. Am. Soc. Nephrol..

[61] Heng J., Li Z.T., Yao D.D., Li X.Y., Cui M.X., Li Z.L. (2026). Anemia in cardiorenal disease: From pathogenesis to treatment. Eur. J. Intern Med..

[62] Vidas M.M., Portolés J., Cobo M., Gorriz J.L., Nuñez J., Cases A. (2025). Anemia Management in the Cardiorenal Patient: A Nephrological Perspective. J. Am. Heart Assoc..

[63] Foley R.N., Parfrey P.S., Harnett J.D., Kent G.M., Murray D.C., Barre P.E. (1996). The impact of anemia on cardiomyopathy, morbidity, and and mortality in end-stage renal disease. Am. J. Kidney Dis..

[64] Carullo N., Sorbo D., Faga T., Pugliese S., Zicarelli M.T., Costa D., Ielapi N., Battaglia Y., Pisani A., Coppolino G. (2024). Anemia and Mineral Bone Disorder in Kidney Disease Patients: The Role of FGF-23 and Other Related Factors. Int. J. Mol. Sci..

[65] Fuchs M.A.A., Burke E.J., Latic N., Murray S.L., Li H., Sparks M.A., Abraham D., Zhang H., Rosenberg P., Saleem U. (2025). Fibroblast growth factor 23 and fibroblast growth factor receptor 4 promote cardiac metabolic remodeling in chronic kidney disease. Kidney Int..

[66] Lin C.Y., Hsu Y.J., Hsu S.C., Chen Y., Lee H.S., Lin S.H., Huang S.M., Tsai C.S., Shih C.C. (2015). CB1 cannabinoid receptor antagonist attenuates left ventricular hypertrophy and Akt-mediated cardiac fibrosis in experimental uremia. J. Mol. Cell. Cardiol..

[67] Savira F., Cao L., Wang I., Yang W., Huang K., Hua Y., Jucker B.M., Willette R.N., Huang L., Krum H. (2017). Apoptosis signal-regulating kinase 1 inhibition attenuates cardiac hypertrophy and cardiorenal fibrosis induced by uremic toxins: Implications for cardiorenal syndrome. PLoS ONE.

[68] Rossi M., Campbell K.L., Johnson D.W., Stanton T., Vesey D.A., Coombes J.S., Weston K.S., Hawley C.M., McWhinney B.C., Ungerer J.P. (2014). Protein-bound uremic toxins, inflammation and oxidative stress: A cross-sectional study in stage 3-4 chronic kidney disease. Arch. Med. Res..

[69] Watanabe H., Miyamoto Y., Honda D., Tanaka H., Wu Q., Endo M., Noguchi T., Kadowaki D., Ishima Y., Kotani S. (2013). P-Cresyl sulfate causes renal tubular cell damage by inducing oxidative stress by activation of NADPH oxidase. Kidney Int..

[70] Lekawanvijit S., Kompa A.R., Manabe M., Wang B.H., Langham R.G., Nishijima F., Kelly D.J., Krum H. (2012). Chronic kidney disease-induced cardiac fibrosis is ameliorated by reducing circulating levels of a non-dialysable uremic toxin, indoxyl sulfate. PLoS ONE.

[71] Marthi A., Donovan K., Haynes R., Wheeler D.C., Baigent C., Rooney C.M., Landray M.J., Moe S.M., Yang J., Holland L. (2018). Fibroblast Growth Factor-23 and Risks of Cardiovascular and Noncardiovascular Diseases: A Meta-Analysis. J. Am. Soc. Nephrol..

[72] Vasquez F., Tiscornia C., Lorca-Ponce E., Aicardi V., Vasquez S. (2025). Cardiorenal Syndrome: Molecular Pathways Linking Cardiovascular Dysfunction and Chronic Kidney Disease Progression. Int. J. Mol. Sci..

[73] Damman K., Testani J.M. (2015). The kidney in heart failure: An update. Eur. Heart J..

[74] Horiuchi Y., Wettersten N., van Veldhuisen D.J., Mueller C., Filippatos G., Nowak R., Hogan C., Kontos M.C., Cannon C.M., Müeller G.A. (2022). Decongestion, kidney injury and prognosis in patients with acute heart failure. Int. J. Cardiol..

[75] Ronco C., McCullough P., Anker S.D., Anand I., Aspromonte N., Bagshaw S.M., Bellomo R., Berl T., Bobek I., Cruz D.N. (2010). Cardio-renal syndromes: Report from the consensus conference of the acute dialysis quality initiative. Eur. Heart J..

[76] Khandait H., Sodhi S.S., Khandekar N., Bhattad V.B. (2025). Cardiorenal Syndrome in Heart Failure with Preserved Ejection Fraction: Insights into Pathophysiology and Recent Advances. Cardiorenal Med..

[77] Di Maria A., Siligato R., Bondanelli M., Fabbian F. (2024). Venous Doppler flow patterns, venous congestion, heart disease and renal dysfunction: A complex liaison. World J. Cardiol..

[78] Kramer T., Brinkkoetter P., Rosenkranz S. (2022). Right Heart Function in Cardiorenal Syndrome. Curr. Heart Fail. Rep..

[79] Kazory A., Ronco C., Koratala A. (2024). Cardiorenal Interactions in Acute Heart Failure: Renal Proximal Tubules in the Spotlight. Cardiorenal Med..

[80] Hidaka N., Inoue K., Kato H., Hoshino Y., Koga M., Kinoshita Y., Takashi Y., Makita N., Fukumoto S., Nangaku M. (2024). FGF-23, Left Ventricular Hypertrophy, and Mortality in Patients With CKD: A Revisit With Mediation Analysis. JACC Adv..

[81] Zhao H., Wu D., Gyamfi M.A., Wang P., Luecht C., Pfefferkorn A.M., Ashraf M.I., Kamhieh-Milz J., Witowski J., Dragun D. (2023). Expanded Hemodialysis ameliorates uremia-induced impairment of vasculoprotective KLF2 and concomitant proinflammatory priming of endothelial cells through an ERK/AP1/cFOS-dependent mechanism. Front. Immunol..

[82] Damman K., van Deursen V.M., Navis G., Voors A.A., van Veldhuisen D.J., Hillege H.L. (2009). Increased central venous pressure is associated with impaired renal function and mortality in a broad spectrum of patients with cardiovascular disease. J. Am. Coll. Cardiol..

[83] Gottlieb S.S., Abraham W., Butler J., Forman D.E., Loh E., Massie B.M., O’Connor M.C., Rich M.W., Stevenson L.W., Young J. (2002). The prognostic importance of different definitions of worsening renal function in congestive heart failure. J. Card. Fail..

[84] National Kidney Foundation (2002). K/DOQI clinical practice guidelines for chronic kidney disease: Evaluation, classification, and stratification. Am. J. Kidney Dis..

[85] Kellum J.A., Levin N., Bouman C., Lameire N. (2002). Developing a consensus classification system for acute renal failure. Curr. Opin. Crit. Care.

[86] Sohal S., Uppal D., Mathai S.V., Wats K., Uppal N.N. (2024). Acute Cardiorenal Syndrome: An Update. Cardiol. Rev..

[87] Post E.H., Vincent J.L. (2018). Renal autoregulation and blood pressure management in circulatory shock. Crit. Care.

[88] Nohria A., Hasselblad V., Stebbins A., Pauly D.F., Fonarow G.C., Shah M., Yancy C.W., Califf R.M., Stevenson L.W., Hill J.A. (2008). Cardiorenal interactions: Insights from the ESCAPE trial. J. Am. Coll. Cardiol..

[89] Flores J., Aggül B., Alvarado M., Soliman D., Pena C., Nugent K. (2026). Renal Venous Hypertension and Kidney Dysfunction: Implications for Clinical Practice. Cardiol. Rev..

[90] Vanhentenrijk S., Demirjian S., Tang W.H.W. (2025). Backward Failure Impairs Kidney Perfusion in Acute Heart Failure. Kidney360.

[91] Carlström M., Wilcox C.S., Arendshorst W.J. (2015). Renal autoregulation in health and disease. Physiol. Rev..

[92] He Y., Hu L., Wang L., Zhu D., Bu X., Shi H., Chen L., Ge Y. (2025). Renal vein congestion aggravates renal injury associated with cardiopulmonary bypass. Perfusion.

[93] Shimada S., Hirose T., Takahashi C., Sato E., Kinugasa S., Ohsaki Y., Kisu K., Sato H., Ito S., Mori T. (2018). Pathophysiological and molecular mechanisms involved in renal congestion in a novel rat model. Sci. Rep..

[94] Husain-Syed F., Rangaswami J., Núñez J., Skrzypek S., Jux C., Gröne H.J., Birk H.W. (2024). Histopathology of congestive nephropathy: A case description and literature review. ESC Heart Fail..

[95] Endo A., Hirose T., Sato S., Ito H., Takahashi C., Ishikawa R., Kamada A., Oba-Yabana I., Kimura T., Takahashi K. (2024). Sodium glucose cotransporter 2 inhibitor suppresses renal injury in rats with renal congestion. Hypertens. Res..

[96] Hartupee J., Mann D.L. (2017). Neurohormonal activation in heart failure with reduced ejection fraction. Nat. Rev. Cardiol..

[97] Schrier R.W. (2006). Role of diminished renal function in cardiovascular mortality: Marker or pathogenetic factor?. J. Am. Coll. Cardiol..

[98] Dutta A., Saha S., Bahl A., Mittal A., Basak T. (2023). A comprehensive review of acute cardio-renal syndrome: Need for novel biomarkers. Front. Pharmacol..

[99] Trask-Marino A.L., Marino B., Lancefield T.F., See E.J., May C.N., Booth L.C., Raman J., Lankadeva Y.R. (2025). Renal macro- and microcirculatory perturbations in acute kidney injury and chronic kidney disease associated with heart failure and cardiac surgery. Am. J. Physiol. Ren. Physiol..

[100] Zhao Y., Xiong W., Li C., Zhao R., Lu H., Song S., Zhou Y., Hu Y., Shi B., Ge J. (2023). Hypoxia-induced signaling in the cardiovascular system: Pathogenesis and therapeutic targets. Signal Transduct. Target. Ther..

[101] Li J., Hu Z. (2025). Research progress on damage-associated molecular patterns in acute kidney injury. Front. Immunol..

[102] Ang S.P., Chia J.E., Lee E., Laezzo M., Machchhar R., Patel S., Davidson G., Jaiswal V., Iglesias J. (2025). Genomics and Multi-Omics Perspectives on the Pathogenesis of Cardiorenal Syndrome. Genes.

[103] Wolf M. (2010). Forging forward with 10 burning questions on FGF23 in kidney disease. J. Am. Soc. Nephrol..

[104] Grabner A., Amaral A.P., Schramm K., Singh S., Sloan A., Yanucil C., Li J., Shehadeh L.A., Hare J.M., David V. (2015). Activation of Cardiac Fibroblast Growth Factor Receptor 4 Causes Left Ventricular Hypertrophy. Cell Metab..

[105] Leifheit-Nestler M., Richter B., Basaran M., Nespor J., Vogt I., Alesutan I., Voelkl J., Lang F., Heineke J., Krick S. (2018). Impact of Altered Mineral Metabolism on Pathological Cardiac Remodeling in Elevated Fibroblast Growth Factor 23. Front. Endocrinol..

[106] Deng C., Wu Y. (2025). Vitamin D-Parathyroid Hormone-Fibroblast Growth Factor 23 Axis and Cardiac Remodeling. Am. J. Cardiovasc. Drugs.

[107] Kim A., Parikh M.A., Khalid H., Frishman W.H., Peterson S.J. (2026). Fibroblast Growth Factor-23 and Cardiovascular Disease in Patients With Chronic Kidney Disease. Cardiol. Rev..

[108] González-Lafuente L., Navarro-García J.A., Rodríguez-Sánchez E., Aceves-Ripoll J., Poveda J., Vázquez-Sánchez S., Mercado-García E., Fernández-Velasco M., Kuro O.M., Liaño F. (2022). Interplay between mineral bone disorder and cardiac damage in acute kidney injury: From Ca(2+) mishandling and preventive role of Klotho in mice to its potential mortality prediction in human. Transl. Res..

[109] Milovanova L.Y., Milovanov Y.S., Kudryavtseva D.V., Markina M.M., Milovanova S.Y., Kozlovskaya L.V., Lebedeva M.V., Beketov V.D., Moiseev S.V., Mukhin N.A. (2015). Role of the morphogenetic proteins FGF-23 and Klotho and the glycoprotein sclerostin in the assessment of the risk of cardiovascular diseases and the prognosis of chronic kidney disease. Ter. Arkhiv.

[110] Panneflek J., Barbarawi M., Kakarlapudi Y., Barbarawi Z., Lauzea B., Meraz-Torres M. (2026). Natriuretic Peptides as Multisystem Regulators: From Clinical Biomarkers to Therapeutic Targets in Cardio-immunology. Cardiovasc. Drugs Ther..

[111] de Bold A.J., Borenstein H.B., Veress A.T., Sonnenberg H. (1981). A rapid and potent natriuretic response to intravenous injection of atrial myocardial extract in rats. Life Sci..

[112] Della Corte V., Pacinella G., Todaro F., Pecoraro R., Tuttolomondo A. (2023). The Natriuretic Peptide System: A Single Entity, Pleiotropic Effects. Int. J. Mol. Sci..

[113] Gong Y., Zhao Y., Li Y., Wang Q., Li C., Song K., Liu J., Chen F. (2025). Corin in cardiovascular diseases and stroke. Clin. Chim. Acta.

[114] Bie P. (2018). Natriuretic Peptides and Normal Body Fluid Regulation. Compr. Physiol..

[115] Bełtowski J., Wójcicka G. (2002). Regulation of renal tubular sodium transport by cardiac natriuretic peptides: Two decades of research. Med. Sci. Monit..

[116] Nakagawa Y., Nishikimi T., Kuwahara K. (2019). Atrial and brain natriuretic peptides: Hormones secreted from the heart. Peptides.

[117] Ojrzyńska-Witek N., Marczak M., Mazurkiewicz Ł., Petryka-Mazurkiewicz J., Miłosz-Wieczorek B., Grzybowski J., Śpiewak M. (2022). Role of cardiac magnetic resonance in heart failure of initially unknown etiology: A 10-year observational study. Kardiol. Pol..

[118] McKie P.M., Cataliotti A., Sangaralingham S.J., Ichiki T., Cannone V., Bailey K.R., Redfield M.M., Rodeheffer R.J., Burnett J.C. (2011). Predictive utility of atrial, N-terminal pro-atrial, and N-terminal pro-B-type natriuretic peptides for mortality and cardiovascular events in the general community: A 9-year follow-up study. Mayo Clin. Proc..

[119] Abassi Z., Hamo-Giladi D.B., Kinaneh S., Heyman S.N. (2024). The endocrine basis of the cardio-renal axis: New perspectives regarding corin. Physiol. Rep..

[120] Zoccali C., Mallamaci F., Benedetto F.A., Tripepi G., Parlongo S., Cataliotti A., Cutrupi S., Giacone G., Bellanuova I., Cottini E. (2001). Cardiac natriuretic peptides are related to left ventricular mass and function and predict mortality in dialysis patients. J. Am. Soc. Nephrol..

[121] Rochette L., Dogon G., Zeller M., Cottin Y., Vergely C. (2021). GDF15 and Cardiac Cells: Current Concepts and New Insights. Int. J. Mol. Sci..

[122] Lippi G., Cervellin G. (2014). Risk assessment of post-infarction heart failure. Systematic review on the role of emerging biomarkers. Crit. Rev. Clin. Lab. Sci..

[123] Slika A., Jomaa D., Zaidan S., Yehya R., Al Moussawi S., Sfeir G., Kfoury H., Njeim R., Ziyadeh F., Eid A. (2025). GDF-15 Drives NETosis and Macrophage Polarization in Diabetic Kidney and Cardiac Injury: Dual-Organ Inflammatory Axis and Therapeutic Target: FR-PO0311. J. Am. Soc. Nephrol..

[124] Moreira G.R., Ávila D.X., Candia A.M.D., Scaramussa V.D., Villacorta H. (2025). Growth Differentiation Factor 15 is Correlated With Urinary Markers in Patients with Chronic Heart Failure. Arq. Bras. Cardiol..

[125] Zhou Z., Liu H., Ju H., Chen H., Jin H., Sun M. (2023). Circulating GDF-15 in relation to the progression and prognosis of chronic kidney disease: A systematic review and dose-response meta-analysis. Eur. J. Intern Med..

[126] Gürgöze M.T., van Vark L.C., Baart S.J., Kardys I., Akkerhuis K.M., Manintveld O.C., Postmus D., Hillege H.L., Lesman-Leegte I., Asselbergs F.W. (2023). Multimarker Analysis of Serially Measured GDF-15, NT-proBNP, ST2, GAL-3, cTnI, Creatinine, and Prognosis in Acute Heart Failure. Circ. Heart Fail..

[127] Sakamoto D., Matsuoka Y., Nakatani D., Okada K., Sunaga A., Kida H., Sato T., Kitamura T., Tamaki S., Seo M. (2025). Role and prognostic value of growth differentiation factor 15 in patient of heart failure with preserved ejection fraction: Insights from the PURSUIT-HFpEF registry. Open Heart.

[128] Garg A., Virmani D., Agrawal S., Agarwal C., Sharma A., Stefanini G., Kostis J.B. (2017). Clinical Application of Biomarkers in Heart Failure with a Preserved Ejection Fraction: A Review. Cardiology.

[129] Benes J., Kotrc M., Wohlfahrt P., Conrad M.J., Franekova J., Jabor A., Lupinek P., Kautzner J., Melenovsky V., Jarolim P. (2019). The Role of GDF-15 in Heart Failure Patients With Chronic Kidney Disease. Can. J. Cardiol..

[130] Song R., Peng W., Zhang Y., Lv F., Wu H.K., Guo J., Cao Y., Pi Y., Zhang X., Jin L. (2013). Central role of E3 ubiquitin ligase MG53 in insulin resistance and metabolic disorders. Nature.

[131] Li H., Duann P., Li Z., Zhou X., Ma J., Rovin B.H., Lin P.H. (2022). The cell membrane repair protein MG53 modulates transcription factor NF-κB signaling to control kidney fibrosis. Kidney Int..

[132] Hayakawa S., Ohashi K., Shibata R., Kataoka Y., Miyabe M., Enomoto T., Joki Y., Shimizu Y., Kambara T., Uemura Y. (2015). Cardiac myocyte-derived follistatin-like 1 prevents renal injury in a subtotal nephrectomy model. J. Am. Soc. Nephrol..

[133] Wang C., Zhou J., Jia P., Yang Y., Song R., Zheng X., Zhang H., Li Y. (2025). Joint proteomic and metabolomic analysis reveals renal metabolic remodeling of chronic heart failure mice. J. Pharm. BioMed Anal..

[134] Li X., Raisinghani N., Gallinat A., Santos-Gallego C.G., Zhang S., La Salvia S., Yoon S., Yavuz H., Phan A., Shao A. (2026). Circulating Extracellular Vesicles in the Pathogenesis of Heart Failure in Patients With Chronic Kidney Disease. Circulation.

[135] Benjamin J.I., Pollock D.M. (2024). Current perspective on circadian function of the kidney. Am. J. Physiol. Ren. Physiol..

[136] Juffre A., Gumz M.L. (2024). Recent advances in understanding the kidney circadian clock mechanism. Am. J. Physiol. Ren. Physiol..

[137] Lecacheur M., Ammerlaan D.J.M., Dierickx P. (2024). Circadian rhythms in cardiovascular (dys)function: Approaches for future therapeutics. npj Cardiovasc. Health.

[138] Rahman A., Hasan A.U., Nishiyama A., Kobori H. (2018). Altered Circadian Timing System-Mediated Non-Dipping Pattern of Blood Pressure and Associated Cardiovascular Disorders in Metabolic and Kidney Diseases. Int. J. Mol. Sci..

[139] Schmitt E.E., Johnson E.C., Yusifova M., Bruns D.R. (2019). The renal molecular clock: Broken by aging and restored by exercise. Am. J. Physiol. Ren. Physiol..

[140] Lombardo R., Tubaro A., Burkhard F. (2020). Nocturia: The Complex Role of the Heart, Kidneys, and Bladder. Eur. Urol. Focus.

[141] Aggarwal B., Gao Y., Alfini A., Azarbarzin A., Anafi R.C., Baron K.G., Bautch V.L., Bowles N., Broussard J.L., Brown M. (2026). Sleep and circadian rhythms in cardiovascular resilience: Mechanisms, implications, and a Roadmap for research and interventions. Nat. Rev. Cardiol..

[142] Lair B., Lac M., Frassin L., Brunet M., Buléon M., Feuillet G., Maslo C., Marquès M., Monbrun L., Bourlier V. (2024). Common mouse models of chronic kidney disease are not associated with cachexia. Commun. Biol..

[143] Pilz P.M., Ward J.E., Chang W.T., Kiss A., Bateh E., Jha A., Fisch S., Podesser B.K., Liao R. (2022). Large and Small Animal Models of Heart Failure With Reduced Ejection Fraction. Circ. Res..

[144] Carll A.P., Willis M.S., Lust R.M., Costa D.L., Farraj A.K. (2011). Merits of non-invasive rat models of left ventricular heart failure. Cardiovasc. Toxicol..

[145] Capone F., Vacca A., Bidault G., Sarver D., Kaminska D., Strocchi S., Vidal-Puig A., Greco C.M., Lusis A.J., Schiattarella G.G. (2025). Decoding the Liver-Heart Axis in Cardiometabolic Diseases. Circ. Res..

[146] Kp M.M.J., Cong S., Yu F., Lim S.Y.R., Yap F.H.X., Wong P., Ramachandra C.J., Hausenloy D.J. (2025). Two-Hit Swine Model of Heart Failure With Preserved Ejection Fraction Mimics Cardiac and Systemic Pathologies. JACC Asia.

[147] Juguilon C., Khosravi R., Radisic M., Wu J.C. (2025). In Vitro Modeling of Interorgan Crosstalk: Multi-Organ-on-a-Chip for Studying Cardiovascular-Kidney-Metabolic Syndrome. Circ. Res..

[148] Gabbin B., Gallant J., Liu F., Mei H., van Meer B.J., Rabelink T.J., Mummery C.L., Vanslambrouck J.M., van den Berg C.W., Meraviglia V. (2026). In vitro modeling of renal injury-induced cardiac effects using human iPSC-derived organoids. Cell Commun. Signal.

[149] Zhang C., Wu Z., Song Y., Jin X., Hu J., Huang C., Zhou J., Lian J. (2024). Exploring diagnostic biomarkers of type 2 cardio-renal syndrome based on secreted proteins and bioinformatics analysis. Sci. Rep..

[150] Gunnarsson S., Vito O., Unwin R.J. (2026). Cardiovascular-kidney-metabolic syndrome: Prevalence, risks, disease trajectories, and early-stage management. Am. J. Physiol. Cell Physiol..

[151] Samir S.M., Hassan H.M., Elmowafy R., Ebrahim N.A., Albadawi E.A., Albadrani M., Owaydhah W.H., Elhadidy M.G. (2025). Adjunctive effects of intermittent fasting and exercise with glibenclamide on diabetic nephropathy in rats: A potential role of the polyol pathway. Front. Physiol..

[152] Moro T., Tinsley G., Pacelli F.Q., Marcolin G., Bianco A., Paoli A. (2021). Twelve Months of Time-restricted Eating and Resistance Training Improves Inflammatory Markers and Cardiometabolic Risk Factors. Med. Sci. Sports Exerc..

[153] Santulli G. (2025). The 2025 AHA/ACC hypertension guidelines: Implications for cardiovascular and renal risk in patients with diabetes. Cardiovasc. Diabetol. Endocrinol. Rep..

[154] O’Hara D.V., Lam C.S.P., McMurray J.J.V., Yi T.W., Hocking S., Dawson J., Raichand S., Januszewski A.S., Jardine M.J. (2024). Applications of SGLT2 inhibitors beyond glycaemic control. Nat. Rev. Nephrol..

[155] Piperis C., Marathonitis A., Anastasiou A., Theofilis P., Mourouzis K., Giannakodimos A., Tryfou E., Oikonomou E., Siasos G., Tousoulis D. (2024). Multifaceted Impact of SGLT2 Inhibitors in Heart Failure Patients: Exploring Diverse Mechanisms of Action. Biomedicines.

[156] Patel S.M., Kang Y.M., Im K., Neuen B.L., Anker S.D., Bhatt D.L., Butler J., Cherney D.Z.I., Claggett B.L., Fletcher R.A. (2024). Sodium-Glucose Cotransporter-2 Inhibitors and Major Adverse Cardiovascular Outcomes: A SMART-C Collaborative Meta-Analysis. Circulation.

[157] Baigent C., Emberson J., Haynes R., Herrington W.G., Judge P., Landray M.J., Mayne K.J., Ng S.Y.A., Preiss D., Roddick A.J. (2022). Impact of diabetes on the effects of sodium glucose co-transporter-2 inhibitors on kidney outcomes: Collaborative meta-analysis of large placebo-controlled trials. Lancet.

[158] McMurray J.J.V., Solomon S.D., Inzucchi S.E., Køber L., Kosiborod M.N., Martinez F.A., Ponikowski P., Sabatine M.S., Anand I.S., Bělohlávek J. (2019). Dapagliflozin in Patients with Heart Failure and Reduced Ejection Fraction. N. Engl. J. Med..

[159] Packer M., Anker S.D., Butler J., Filippatos G., Pocock S.J., Carson P., Januzzi J., Verma S., Tsutsui H., Brueckmann M. (2020). Zannad, Cardiovascular and Renal Outcomes with Empagliflozin in Heart Failure. N. Engl. J. Med..

[160] Anker S.D., Butler J., Filippatos G., Ferreira J.P., Bocchi E., Böhm M., Brunner-La Rocca H.P., Choi D.J., Chopra V., Chuquiure-Valenzuela E. (2021). Empagliflozin in Heart Failure with a Preserved Ejection Fraction. N. Engl. J. Med..

[161] Solomon S.D., McMurray J.J.V., Claggett B., de Boer R.A., DeMets D., Hernandez A.F., Inzucchi S.E., Kosiborod M.N., Lam C.S.P., Martinez F. (2022). Dapagliflozin in Heart Failure with Mildly Reduced or Preserved Ejection Fraction. N. Engl. J. Med..

[162] Marso S.P., Daniels G.H., Brown-Frandsen K., Kristensen P., Mann J.F., Nauck M.A., Nissen S.E., Pocock S., Poulter N.R., Ravn L.S. (2016). Liraglutide and Cardiovascular Outcomes in Type 2 Diabetes. N. Engl. J. Med..

[163] Hosseinpour A., Sood A., Kamalpour J., Zandi E., Pakmehr S., Hosseinpour H., Sood A., Agrawal A., Gupta R. (2024). Glucagon-Like Peptide-1 Receptor Agonists and Major Adverse Cardiovascular Events in Patients With and Without Diabetes: A Meta-Analysis of Randomized-Controlled Trials. Clin. Cardiol..

[164] Singh K., Frishman W.H. (2025). Beyond Glycemic Control: Glucagon-Like Peptide-1 Receptor Agonists as Potential Modifiers of Aortic Disease. Cardiol. Rev..

[165] Marso S.P., Bain S.C., Consoli A., Eliaschewitz F.G., Jódar E., Leiter L.A., Lingvay I., Rosenstock J., Seufert J., Warren M.L. (2016). Semaglutide and Cardiovascular Outcomes in Patients with Type 2 Diabetes. N. Engl. J. Med..

[166] Sattar N., Lee M.M.Y., Kristensen S.L., Branch K.R.H., Del Prato S., Khurmi N.S., Lam C.S.P., Lopes R.D., McMurray J.J.V., Pratley R.E. (2021). Cardiovascular, mortality, and kidney outcomes with GLP-1 receptor agonists in patients with type 2 diabetes: A systematic review and meta-analysis of randomised trials. Lancet Diabetes Endocrinol..

[167] Perkovic V., Tuttle K.R., Rossing P., Mahaffey K.W., Mann J.F.E., Bakris G., Baeres F.M.M., Idorn T., Bosch-Traberg H., Lausvig N.L. (2024). Effects of Semaglutide on Chronic Kidney Disease in Patients with Type 2 Diabetes. N. Engl. J. Med..

[168] Jhund P.S., Talebi A., Henderson A.D., Claggett B.L., Vaduganathan M., Desai A.S., Lam C.S.P., Pitt B., Senni M., Shah S.J. (2024). Mineralocorticoid receptor antagonists in heart failure: An individual patient level meta-analysis. Lancet.

[169] Pitt B., Pfeffer M.A., Assmann S.F., Boineau R., Anand I.S., Claggett B., Clausell N., Desai A.S., Diaz R., Fleg J.L. (2014). Spironolactone for heart failure with preserved ejection fraction. N. Engl. J. Med..

[170] Amazit L., Le Billan F., Kolkhof P., Lamribet K., Viengchareun S., Fay M.R., Khan J.A., Hillisch A., Lombès M., Rafestin-Oblin M.E. (2015). Finerenone Impedes Aldosterone-dependent Nuclear Import of the Mineralocorticoid Receptor and Prevents Genomic Recruitment of Steroid Receptor Coactivator-1. J. Biol. Chem..

[171] Agarwal R., Kolkhof P., Bakris G., Bauersachs J., Haller H., Wada T., Zannad F. (2021). Steroidal and non-steroidal mineralocorticoid receptor antagonists in cardiorenal medicine. Eur. Heart J..

[172] Bakris G.L., Agarwal R., Anker S.D., Pitt B., Ruilope L.M., Rossing P., Kolkhof P., Nowack C., Schloemer P., Joseph A. (2020). Effect of Finerenone on Chronic Kidney Disease Outcomes in Type 2 Diabetes. N. Engl. J. Med..

[173] Pitt B., Filippatos G., Agarwal R., Anker S.D., Bakris G.L., Rossing P., Joseph A., Kolkhof P., Nowack C., Schloemer P. (2021). Cardiovascular Events with Finerenone in Kidney Disease and Type 2 Diabetes. N. Engl. J. Med..

[174] Solomon S.D., McMurray J.J.V., Vaduganathan M., Claggett B., Jhund P.S., Desai A.S., Henderson A.D., Lam C.S.P., Pitt B., Senni M. (2024). Finerenone in Heart Failure with Mildly Reduced or Preserved Ejection Fraction. N. Engl. J. Med..

[175] Ostrominski J.W., Claggett B.L., Miao Z.M., Filippatos G., Desai A.S., Jhund P.S., Henderson A., Brinker M., Schloemer P., Viswanathan P. (2025). Efficacy and Safety of Finerenone in Type 2 Diabetes: A Pooled Analysis of Trials of Heart Failure and Chronic Kidney Disease. Diabetes Care.

[176] Agarwal R., Green J.B., Heerspink H.J.L., Mann J.F.E., McGill J.B., Mottl A.K., Nangaku M., Rosenstock J., Vaduganathan M., Brinker M. (2025). Impact of Baseline GLP-1 Receptor Agonist Use on Albuminuria Reduction and Safety With Simultaneous Initiation of Finerenone and Empagliflozin in Type 2 Diabetes and Chronic Kidney Disease (CONFIDENCE Trial). Diabetes Care.

[177] Zhang Y., Jiang L., Wang J., Wang T., Chien C., Huang W., Fu X., Xiao Y., Fu Q., Wang S. (2022). Network meta-analysis on the effects of finerenone versus SGLT2 inhibitors and GLP-1 receptor agonists on cardiovascular and renal outcomes in patients with type 2 diabetes mellitus and chronic kidney disease. Cardiovasc. Diabetol..

[178] Zhao L.M., Zhan Z.L., Ning J., Qiu M. (2021). Network Meta-Analysis on the Effects of SGLT2 Inhibitors Versus Finerenone on Cardiorenal Outcomes in Patients with Type 2 Diabetes and Chronic Kidney Disease. Front. Pharmacol..

